# Supplementary figures and images for: Stress-dependent cell stiffening by tardigrade tolerance proteins that reversibly form a filamentous network and gel
Source: PLoS Biol. 2022 Sep 6;20(9):e3001780. doi: 10.1371/journal.pbio.3001780 (PMC9592077; doi:10.1371/journal.pbio.3001780)

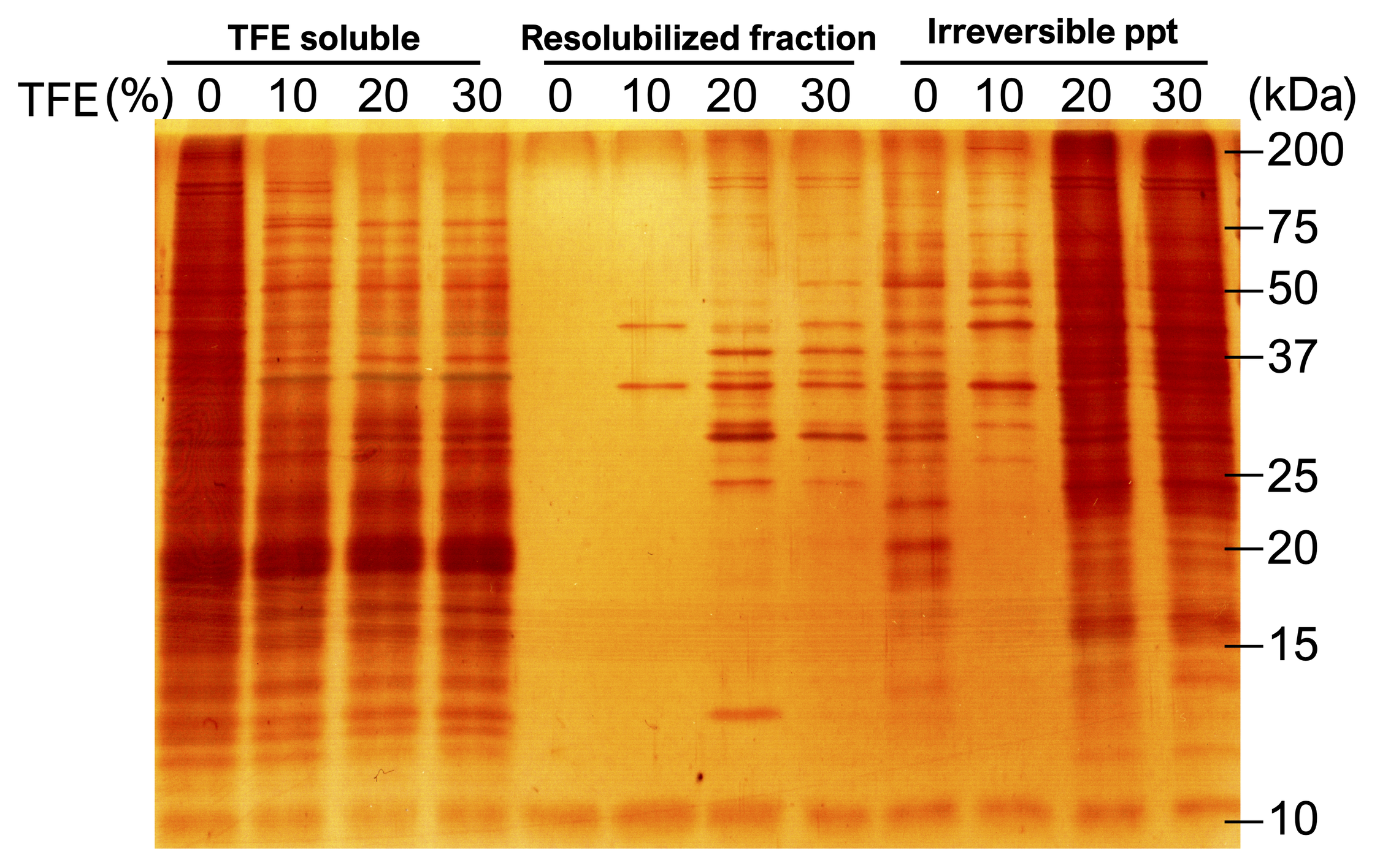

Supplement: S1 Fig — Each fraction was analyzed by SDS-PAGE and visualized by silver-staining. The image of the resolubilized fraction is partly presented in Fig 1B. As the concentration of TFE increased (0% to 20%), proteins decreased in the TFE soluble fraction, and proteins increased in both the irreversible precipitate and the resolubilized fraction. Treatment with 20% and 30% TFE had largely similar effects. (TIF) [file pbio.3001780.s001.tif]

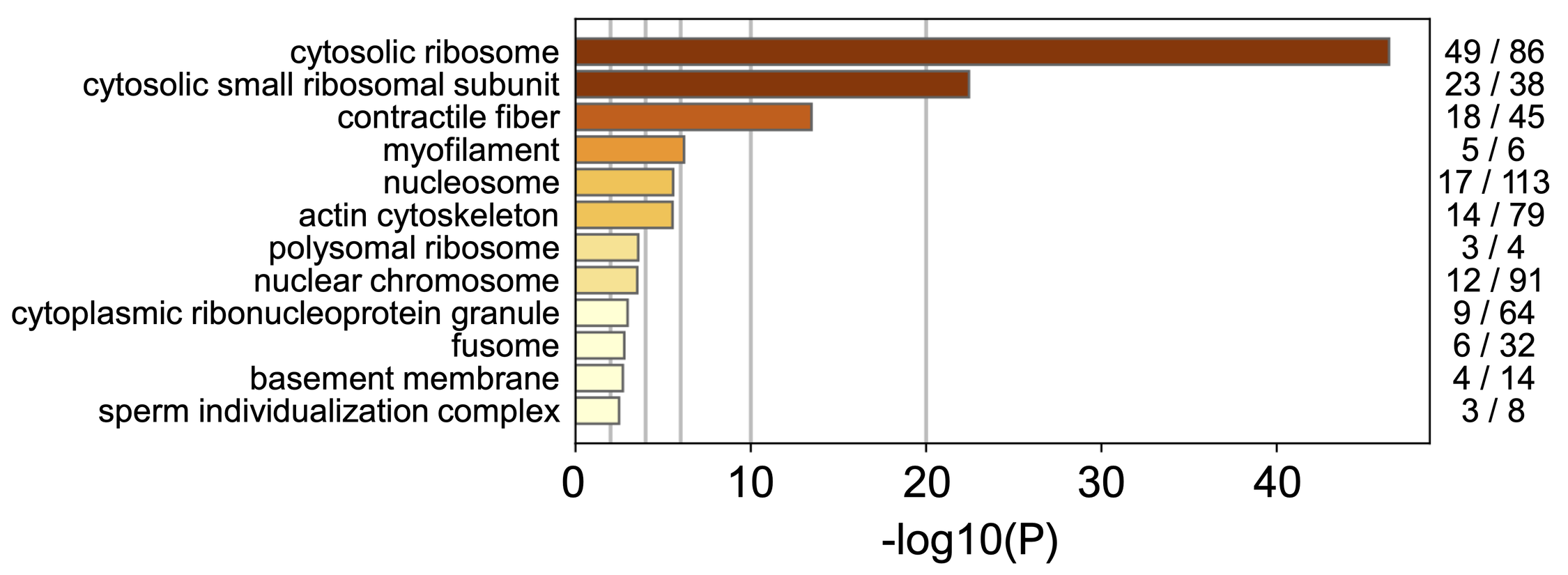

Supplement: S2 Fig — Ribosomal proteins and fiber proteins were highly enriched in T-DRYPs. GO terms of cellular components enriched in T-DRYPs were analyzed by Metascape. The numerical data are available in S4 Data. (TIF) [file pbio.3001780.s002.tif]

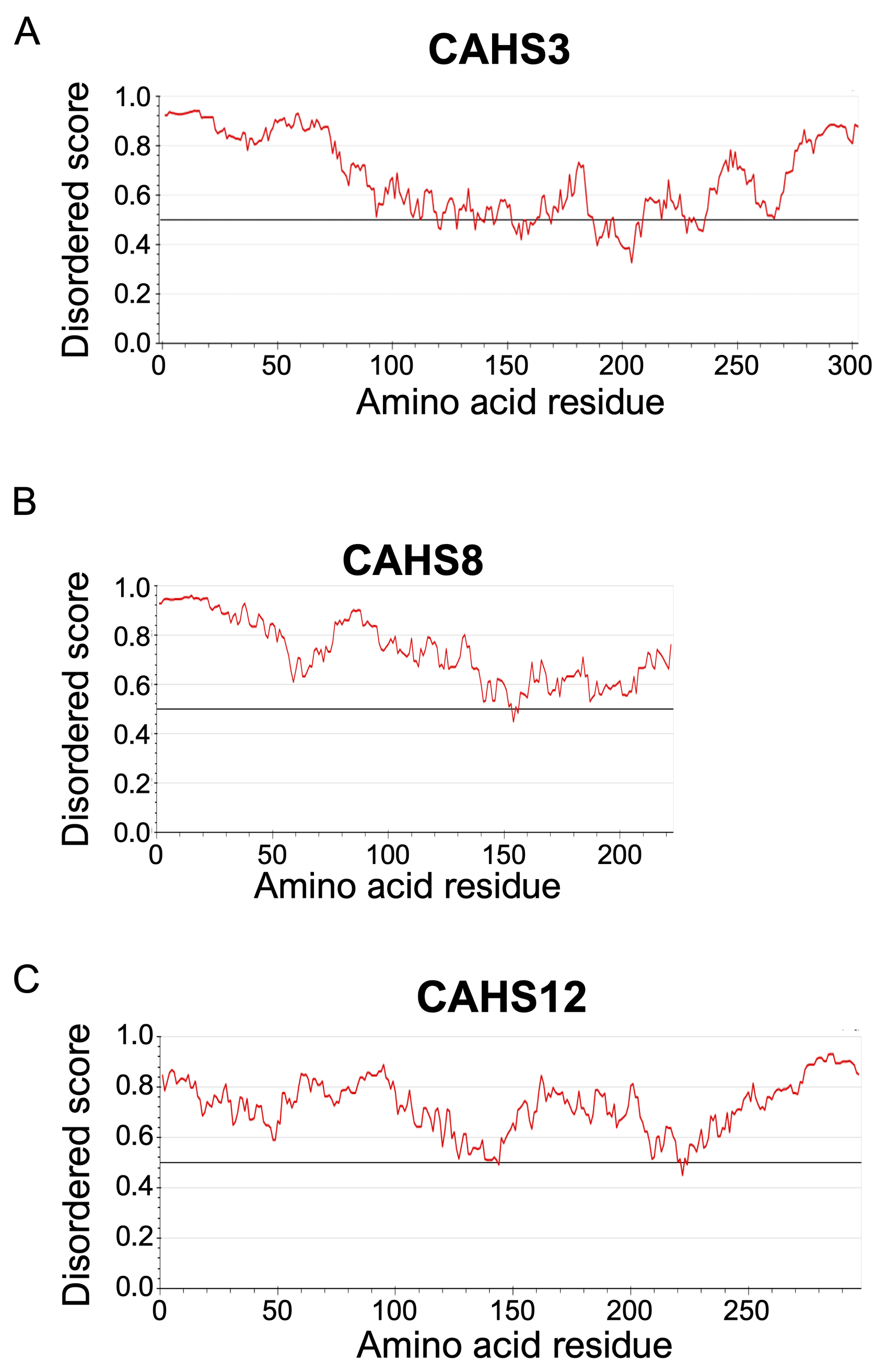

Supplement: S3 Fig — (A–C) The unstructured score of each amino acid residue was calculated by IUPred2A for CAHS3 (A), CAHS8 (B), and CAHS12 (C). Scores above 0.5 indicate that the region is disordered. Each protein was predicted to be largely disordered throughout. The numerical data are available in S4 Data (A–C). (TIF) [file pbio.3001780.s003.tif]

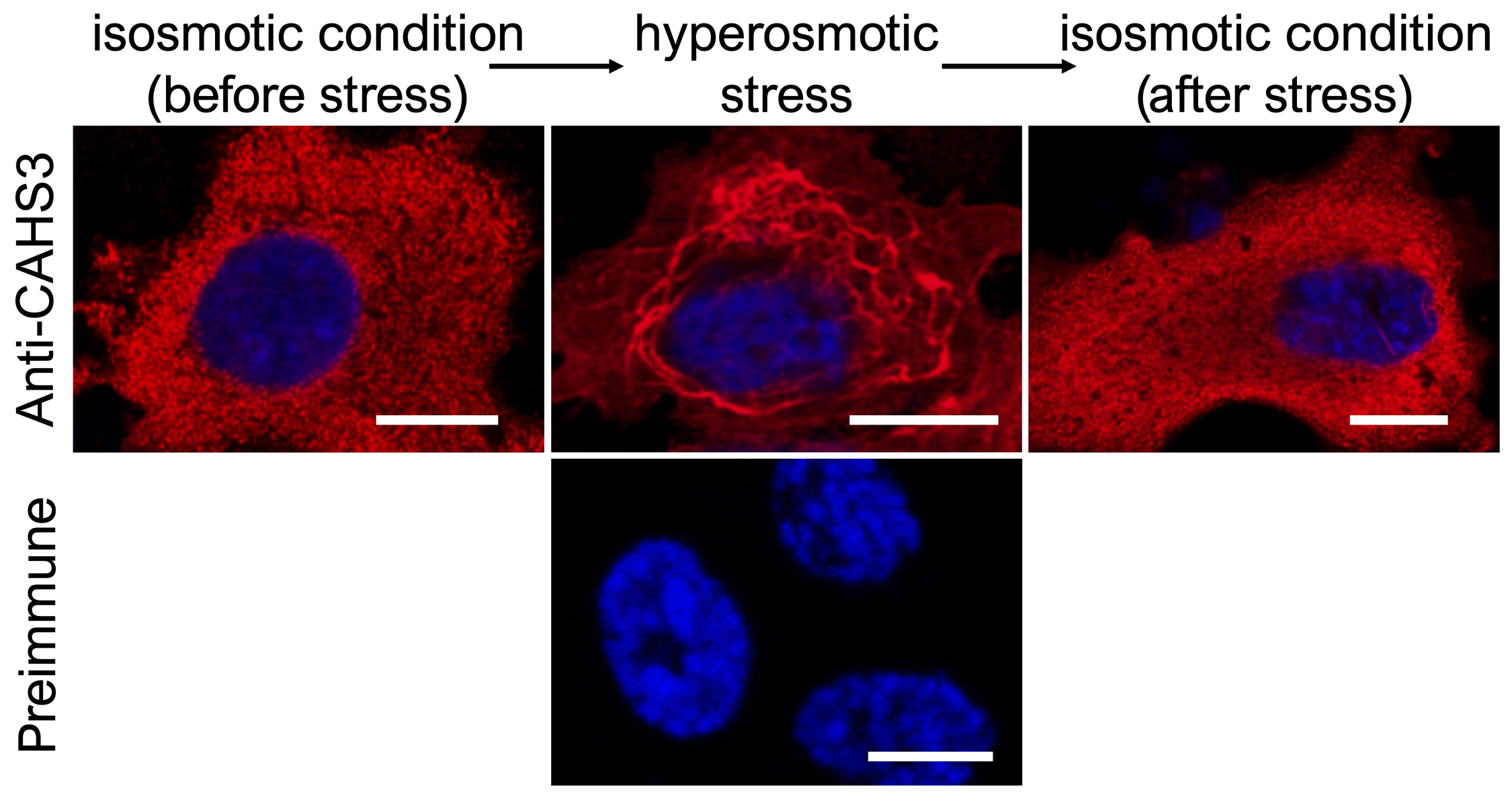

Supplement: S4 Fig — CAHS3 proteins were transiently expressed in HEp-2 cells and detected by immunofluorescence under isosmotic or hyperosmotic conditions. The detected distribution changes were similar to those of GFP-labeled CAHS3. Blue indicates DAPI staining of nuclei. Scale bar, 10 μm. (TIF) [file pbio.3001780.s004.tif]

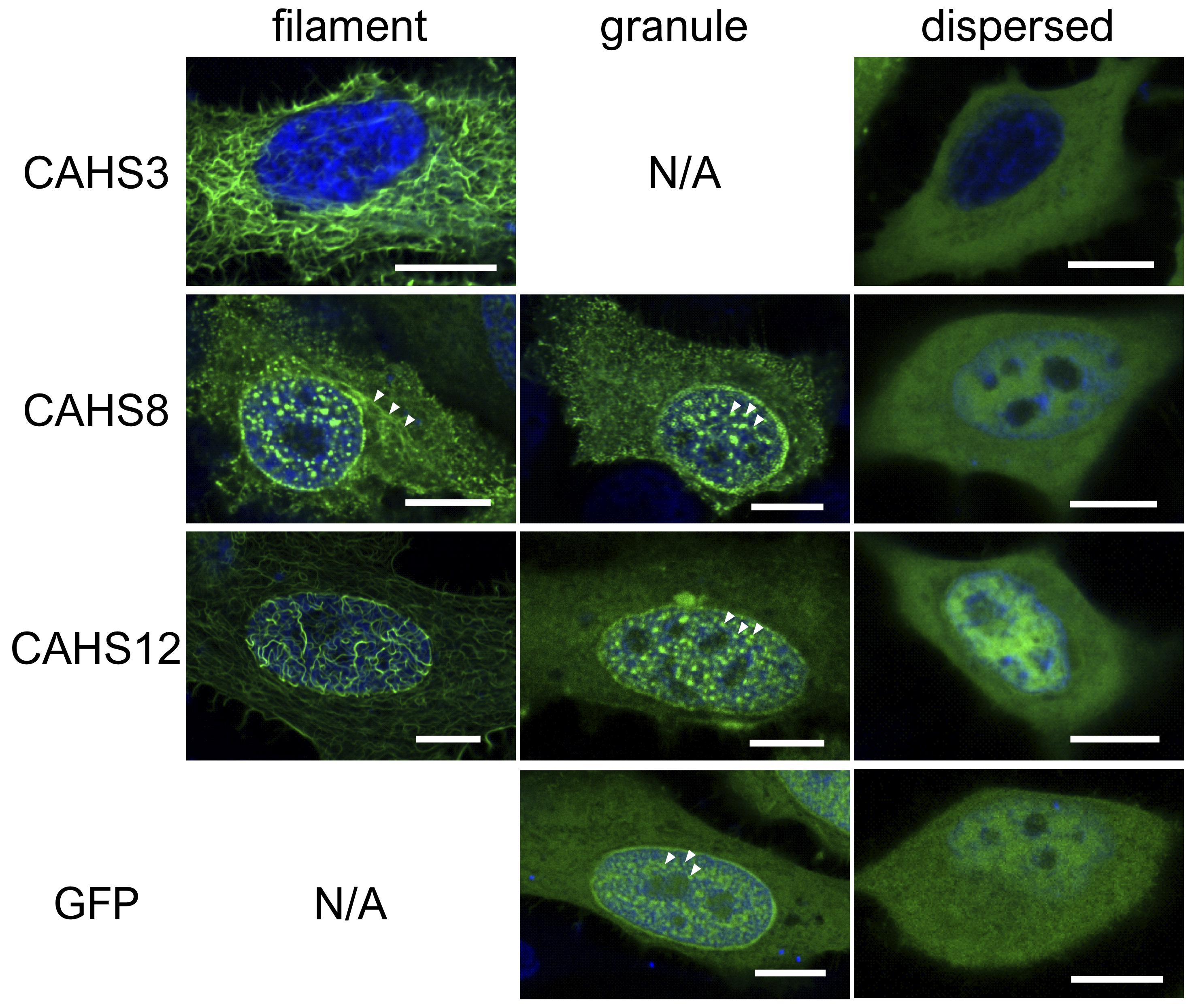

Supplement: S5 Fig — N/A indicates that the corresponding distribution pattern is not or rarely found in a hyperosmotic condition. Scale bar, 10 μm. (TIF) [file pbio.3001780.s005.tif]

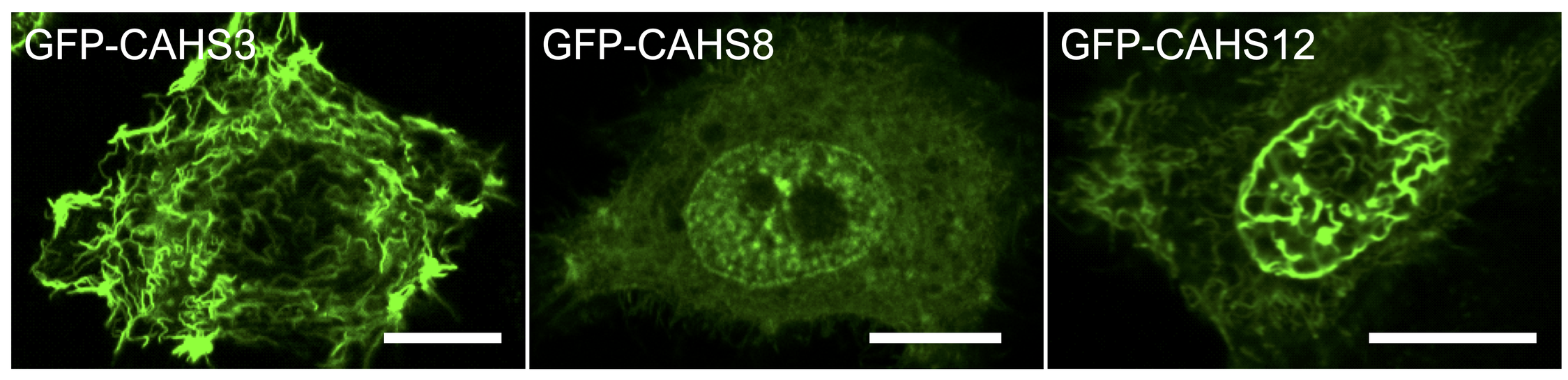

Supplement: S6 Fig — N-terminally GFP-fused CAHS3 and CAHS12 exhibited filament-formation in response to hyperosmotic stress, and CAHS8 formed granule-like condensates like C-terminally GFP-fused CAHS proteins. The GFP-fusion site (N or C-terminus) did not affect the distribution pattern of CAHS proteins. (TIF) [file pbio.3001780.s006.tif]

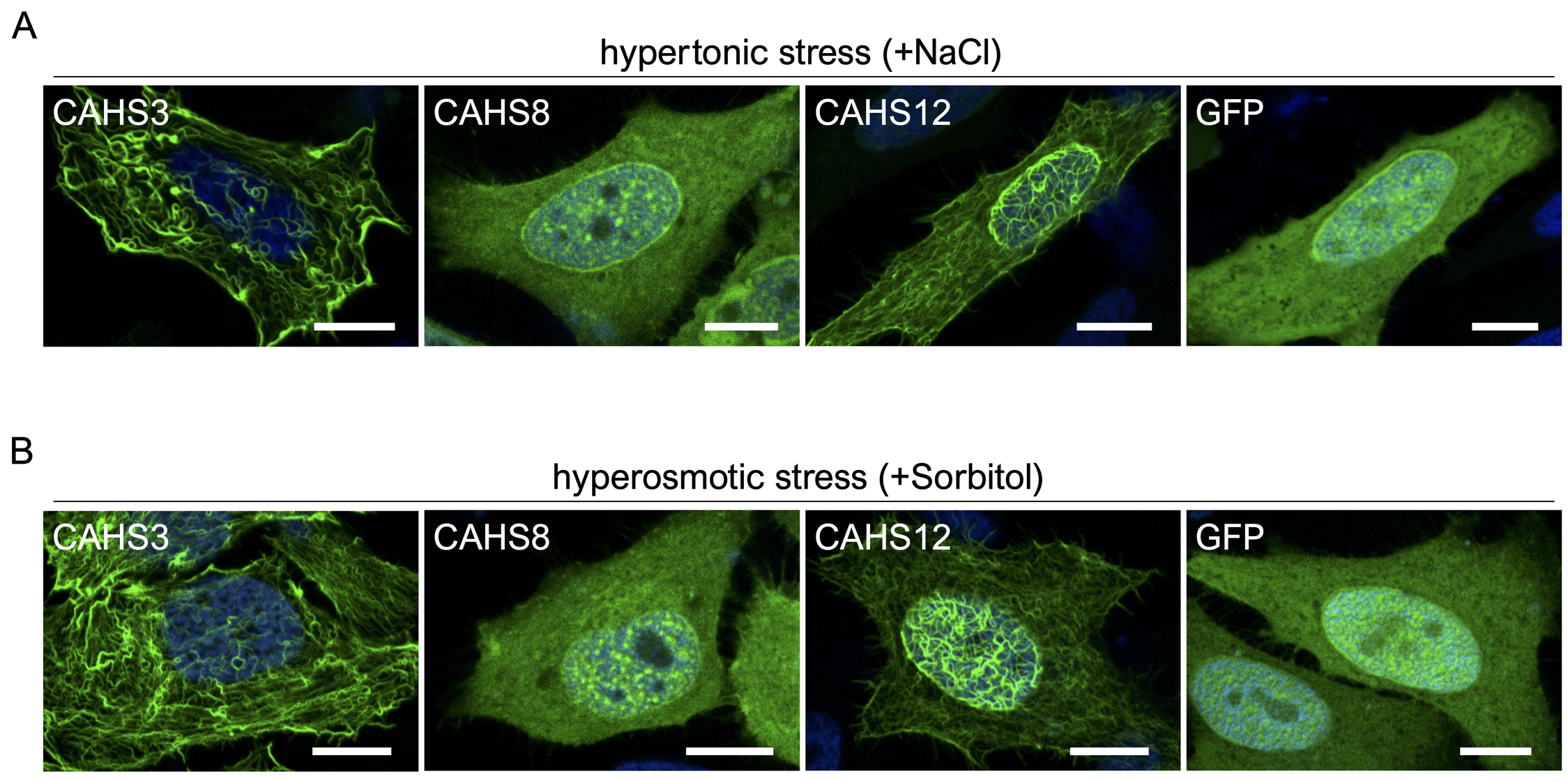

Supplement: S7 Fig — (A–B) Representative distribution patterns of GFP-tagged CAHS proteins and GFP alone under hypertonic medium supplemented with 0.2 M NaCl (A) or hyperosmotic medium supplemented with 0.4 M sorbitol (B). Distribution changes were similar to those observed when treated with 0.4 M trehalose (Fig 2A). Blue indicates Hoechst33342 staining of nuclei. Scale bar, 10 μm. (TIF) [file pbio.3001780.s007.tif]

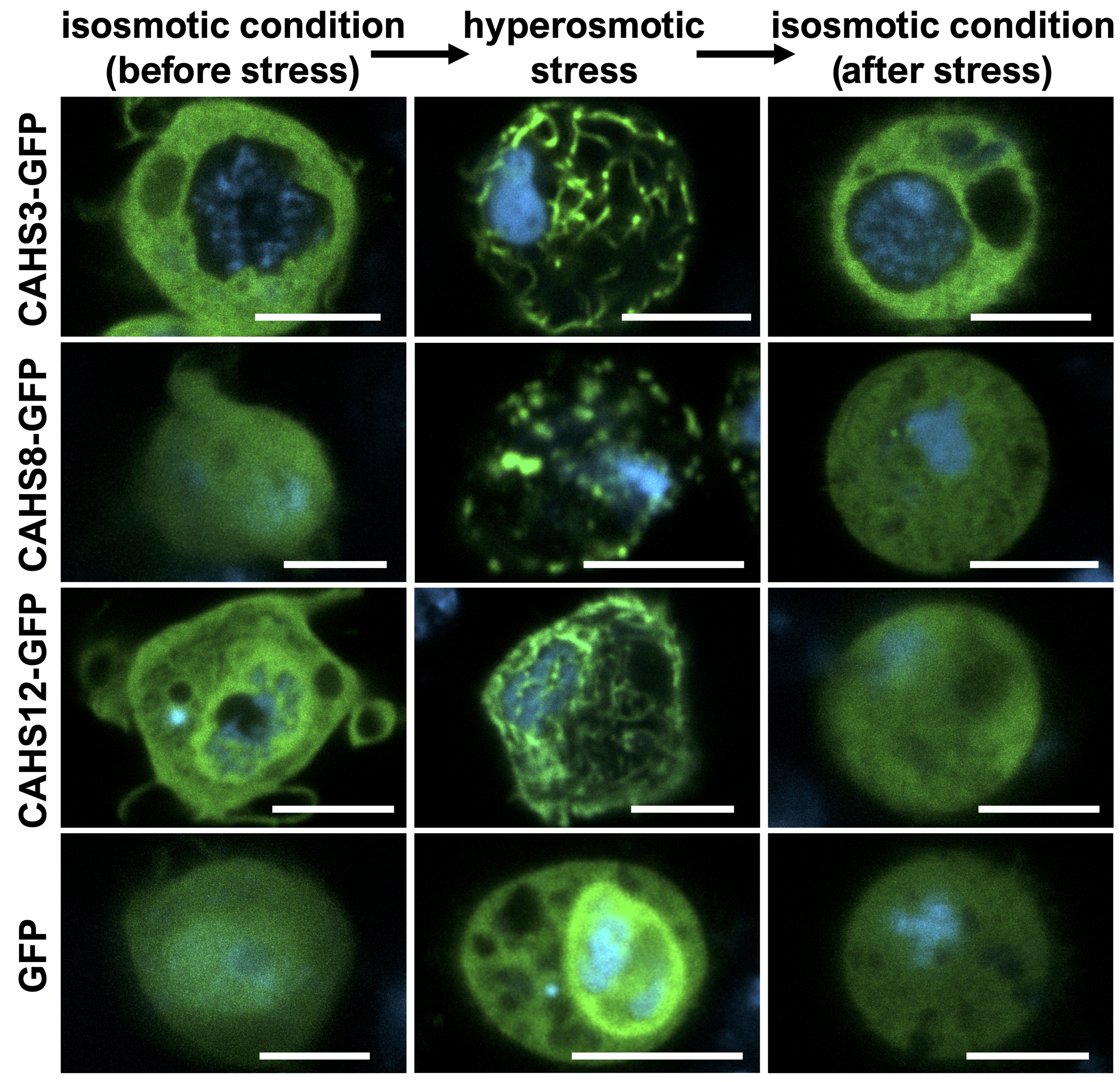

Supplement: S8 Fig — Like in human cells, CAHS3-GFP and CAHS12-GFP reversibly formed filaments and CAHS8-GFP reversibly formed granules upon hyperosmotic stress in fly cells. As a hyperosmotic medium, the culture medium containing 0.4 M trehalose was used. Blue indicates Hoechst33342 staining of nuclei. Scale bar, 5 μm. (TIF) [file pbio.3001780.s008.tif]

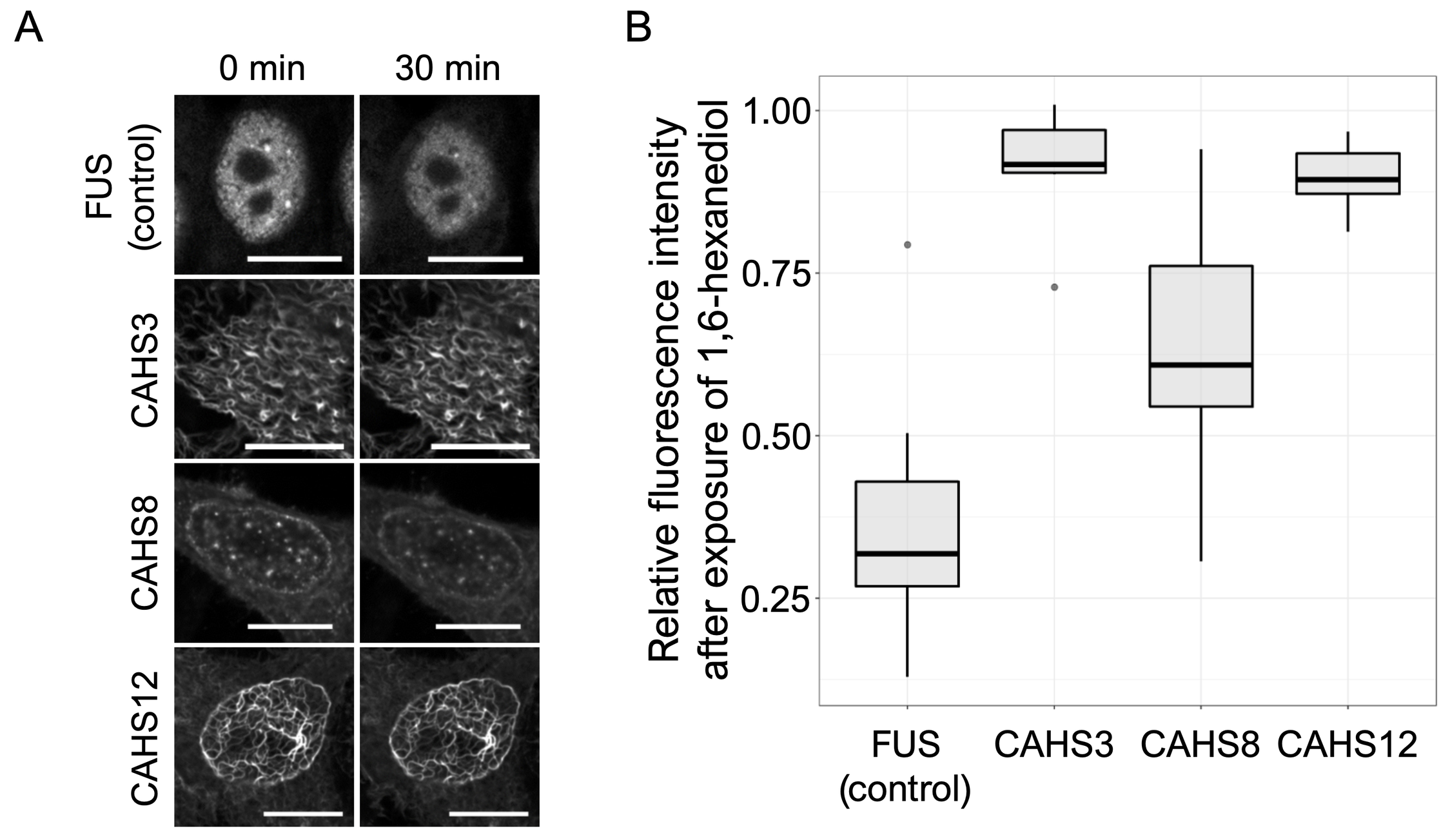

Supplement: S9 Fig — (A) Representative confocal images of human cells expressing FUS-venus (n = 15), CAHS3-GFP (n = 7), CAHS8-GFP (n = 24), or CAHS12-GFP (n = 7) under hyperosmotic and 1,6-hexanediol stress. Exposure to 1,6-hexanediol for 30 min dispersed FUS and CAHS8 condensates. FUS is a control protein sensitive to 1,6-hexanediol. (B) Box plots show the distributions of the fluorescence intensity at 30 min relative to that at 0 min. CAHS3 and CAHS12 filaments were not unaffected by 1,6-hexanediol. Center bar and edges indicate 50th, 25th, and 75th percentiles, respectively, and whiskers correspond to the 1.5 interquartile range. Scale bar, 10 μm. The underlying numerical data are available in S4 Data (B). (TIF) [file pbio.3001780.s009.tif]

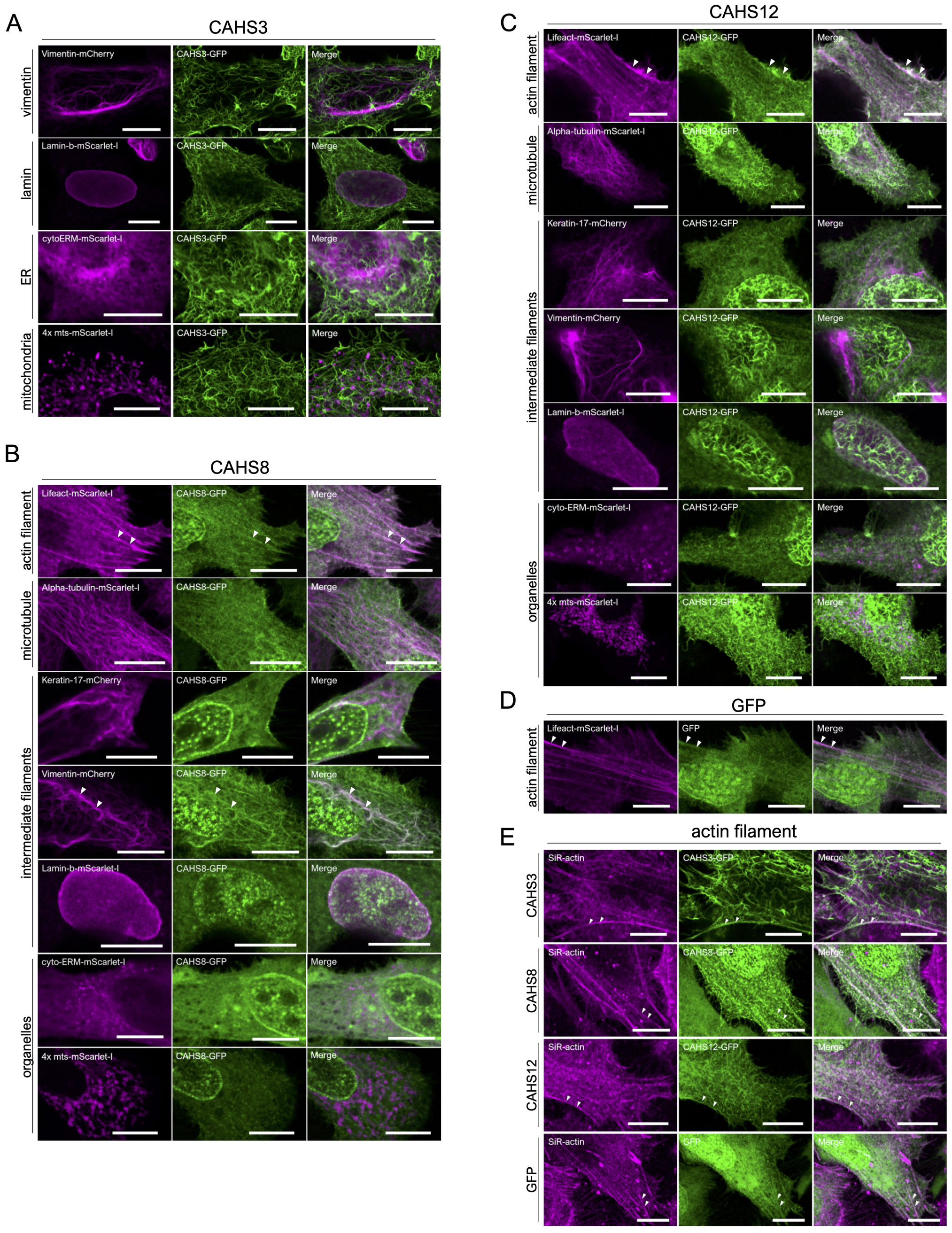

Supplement: S10 Fig — (A–C) Confocal images of HEp-2 cells expressing AcGFP1-tagged CAHS3 (A), CAHS8 (B) or CAHS12 (C) and other fluorescently labeled actin filaments, microtubules, 3 intermediate filaments (keratin, vimentin, and lamin) or 2 organelle markers (endoplasmic reticulum and mitochondria) under hyperosmosis. CAHS filaments or granules did not colocalized with almost all examined intracellular structures, except for vimentin and actin filaments. Although CAHS8 overlapped vimentin filaments (B), tardigrades have no vimentin homologues. White arrowheads indicate detected co-localization. (D and E) Co-localization analyses between intrinsic actin filaments and CAHS-GFP proteins or GFP alone. Actin filaments was visualized by staining with Lifeact-mScarlet-I (D) or the chemical probe SiR-actin (E). All examined GFP-fusion proteins including GFP alone slightly co-localized with actin filaments, suggesting that GFP-moiety causes weak interaction with actin filaments. Scale bar, 10 μm. (TIF) [file pbio.3001780.s010.tif]

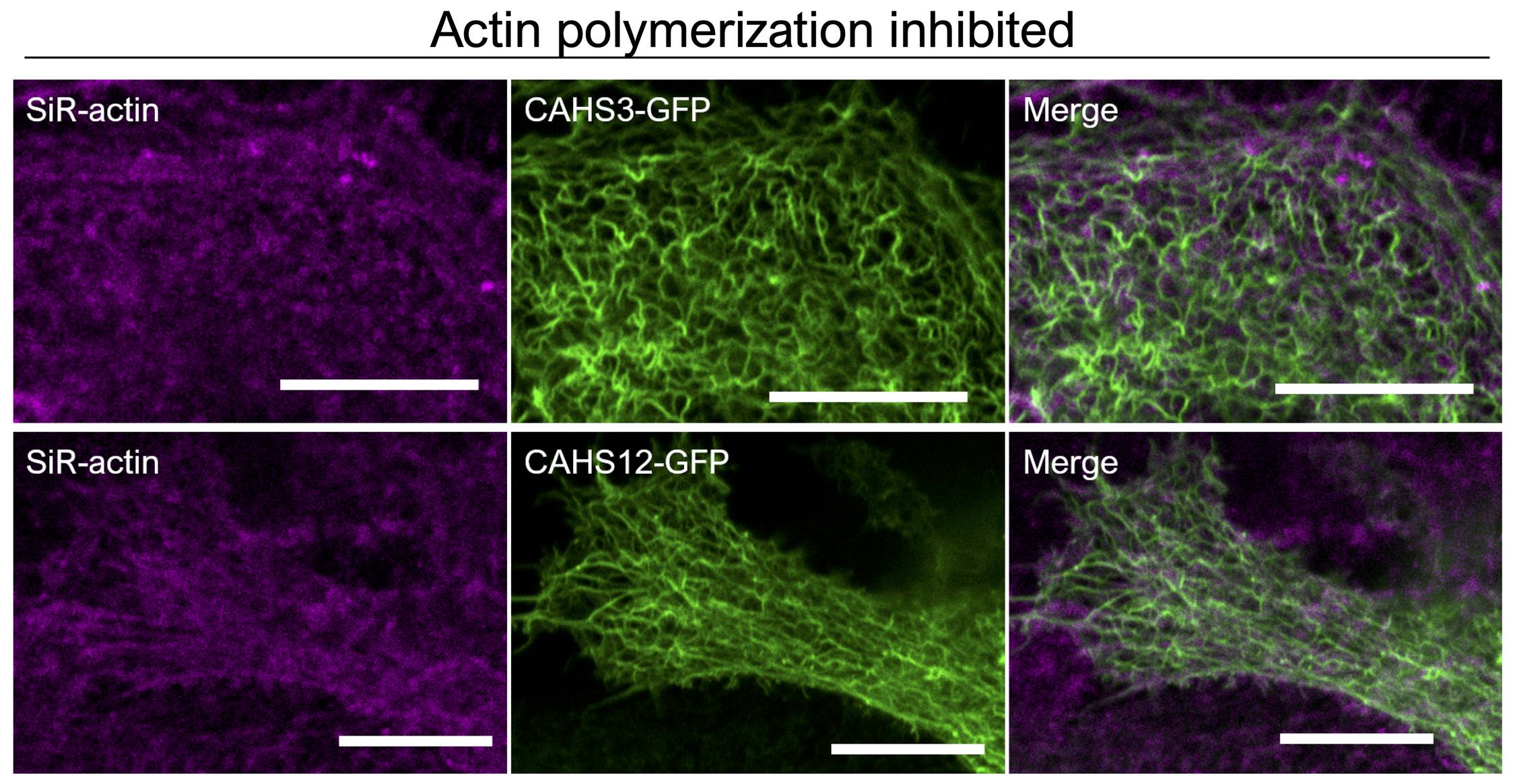

Supplement: S11 Fig — Depolymerization of actin filaments had no effects on the formation of CAHS filaments. Scale bar, 10 μm. (TIF) [file pbio.3001780.s011.tif]

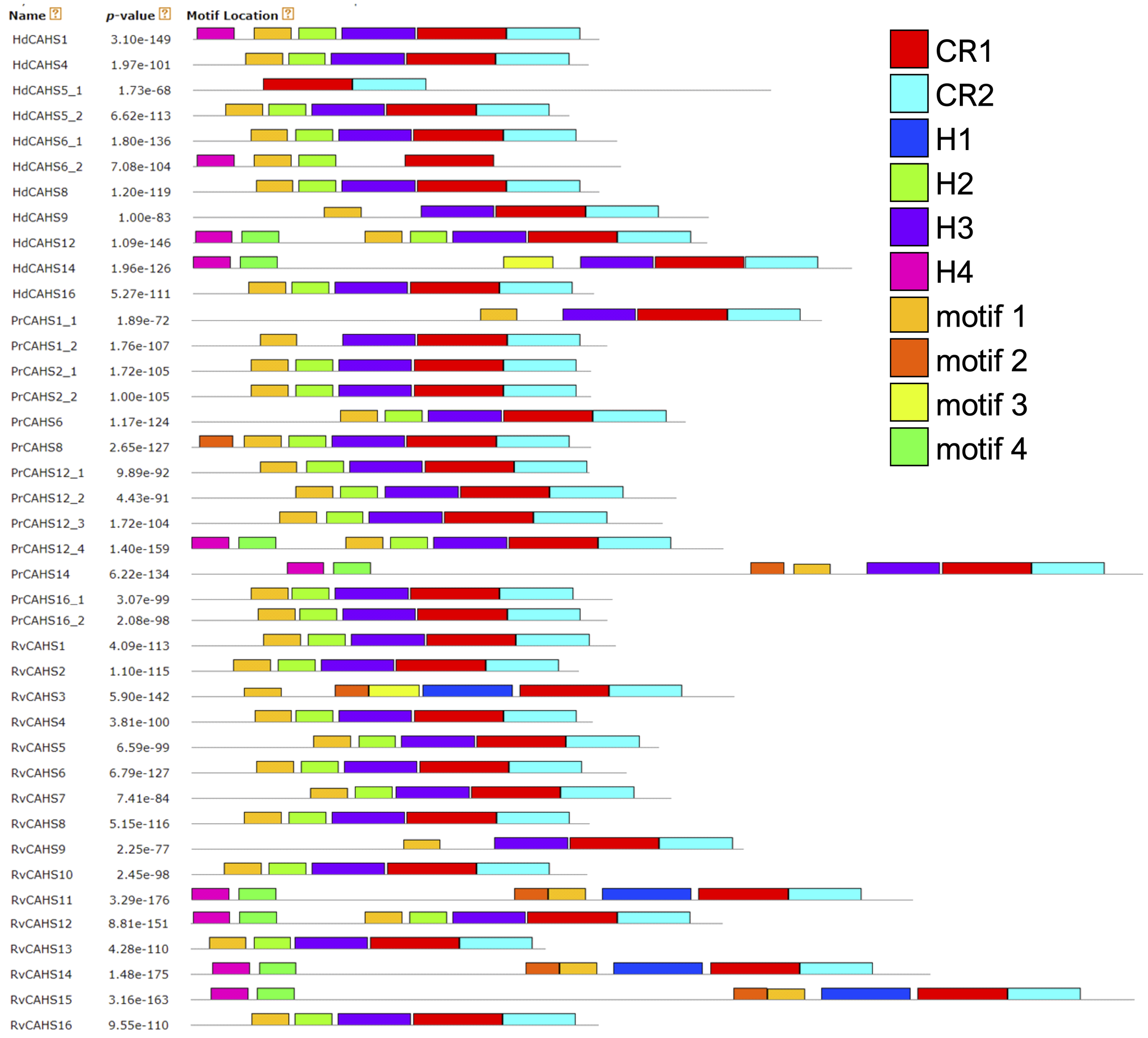

Supplement: S12 Fig — Ten conserved sequence motifs were identified by MEME among 40 CAHS proteins from 3 tolerant tardigrades (Hypsibius exemplaris, Paramacrobiotus metropolitanus, and Ramazzottius varieornatus). Each motif is shown in the corresponding colored box. Both CR1 and CR2 were conserved in all 40 CAHS proteins except CR2 in HdCAHS6_2. CR1, CR2, H1, H2, H3, and H4 were predicted as helical regions by JPred4 (S12 Fig). Hd, H. exemplaris (formerly H. dujardini); Pr, P. metropolitanus (formerly Paramacrobiotus sp. TYO); Rv, R. varieornatus. (TIF) [file pbio.3001780.s012.tif]

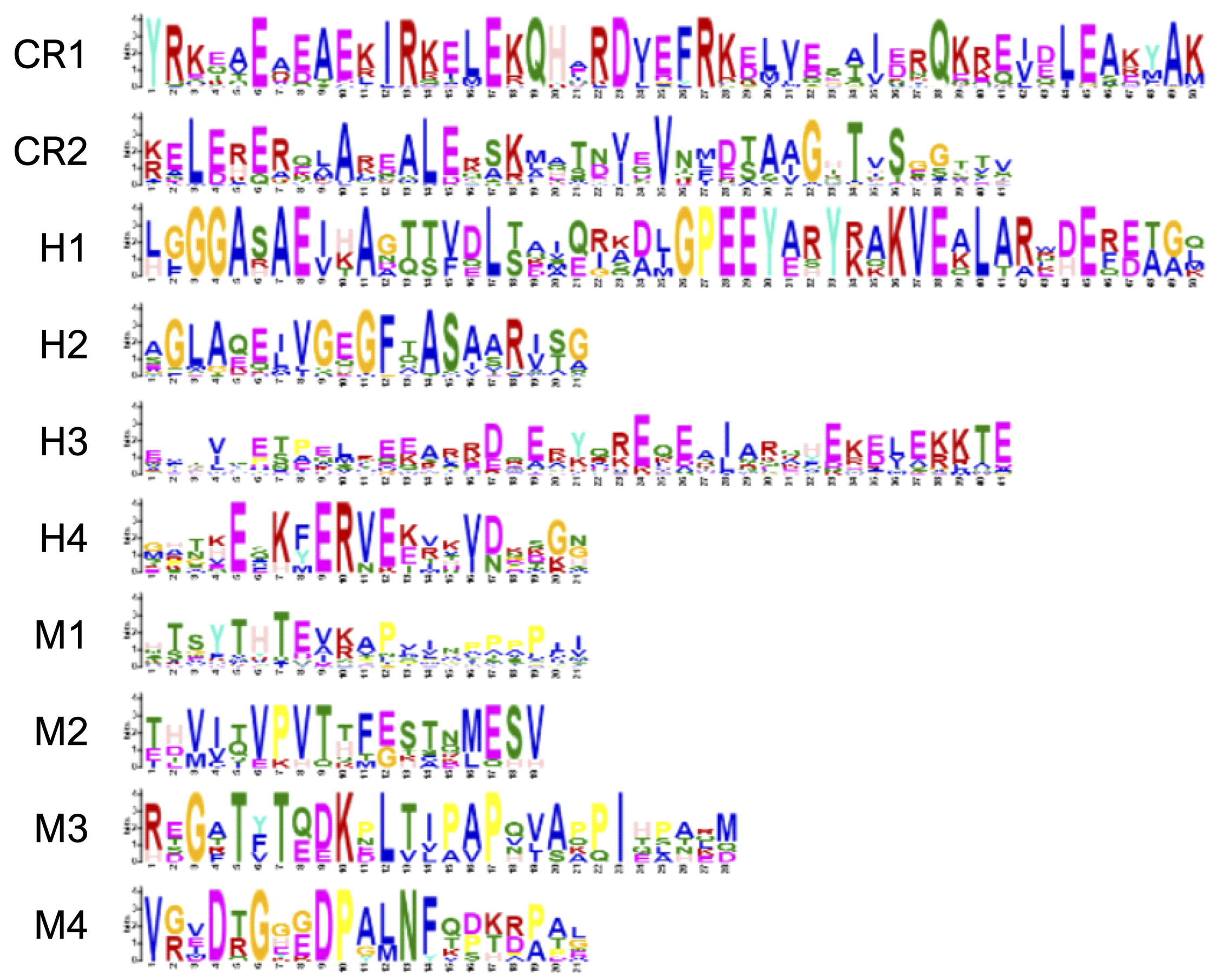

Supplement: S13 Fig — (TIF) [file pbio.3001780.s013.tif]

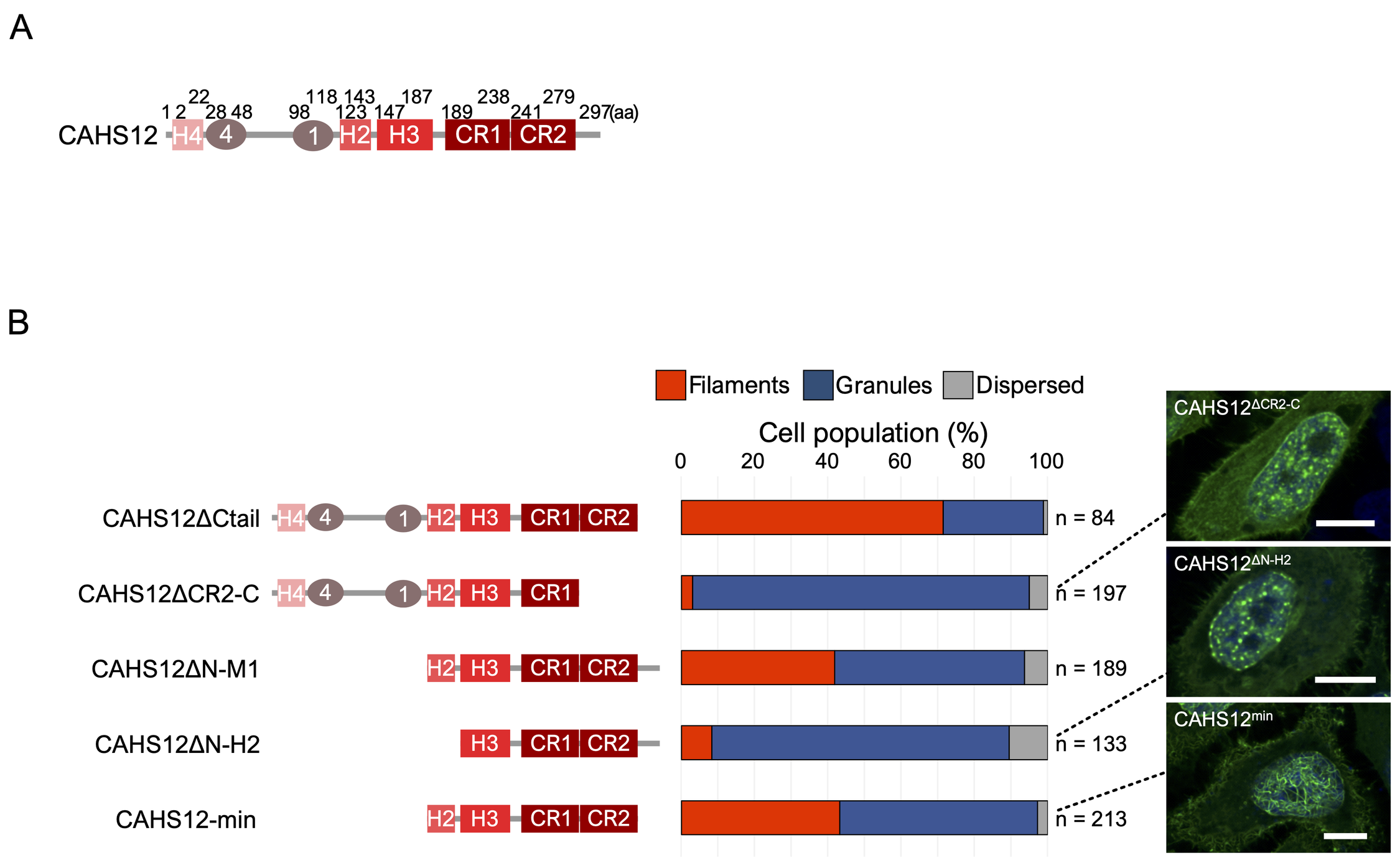

Supplement: S14 Fig — (A) Schematic diagrams of CAHS12 proteins. “CR1” and “CR2” indicate putative helical motifs conserved in CAHS family. “H2,” “H3,” and “H4” indicate putative helical conserved motifs; “1” and “4” indicate other conserved motifs. (B) Schematic diagrams and the corresponding distribution patterns of the CAHS12 truncated mutants. Blue indicates Hoechst33342 staining of nuclei. Scale bar, 10 μm. The underlying numerical data are available in S4 Data (B). (TIF) [file pbio.3001780.s014.tif]

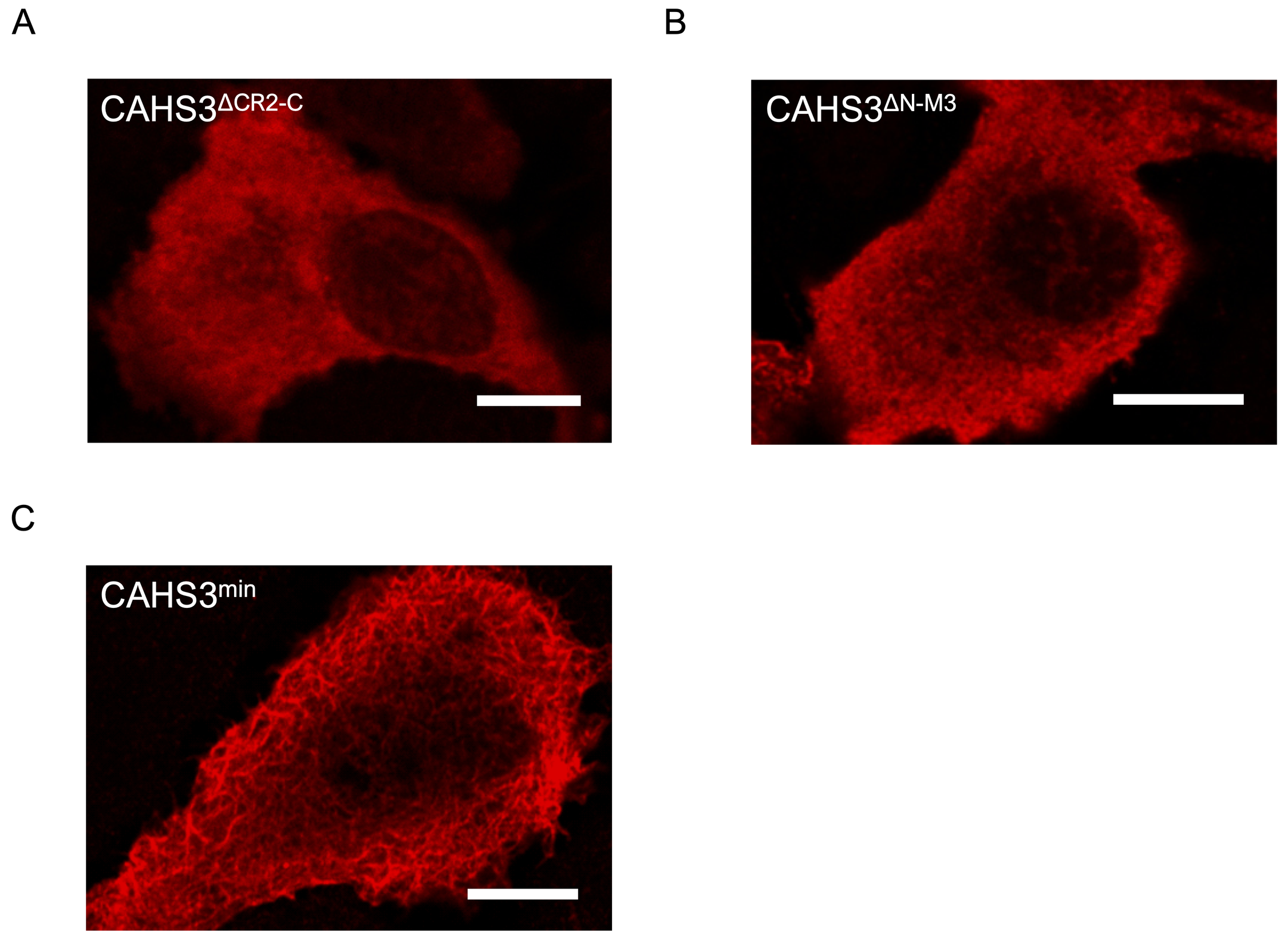

Supplement: S15 Fig — (A–C) CAHS3 truncated mutants were expressed in HEp-2 cells and their distribution patterns were detected by immunofluorescence under a hyperosmotic condition. CAHS3ΔCR2-C (A) and CAHS3ΔN-M3 (B) failed to form long filamentous networks, whereas CAHS3-min (C) successfully formed filaments. The detected distribution patterns were similar to those of the corresponding CAHS3 mutants labeled with GFP (Fig 3B). Scale bar, 10 μm. (TIF) [file pbio.3001780.s015.tif]

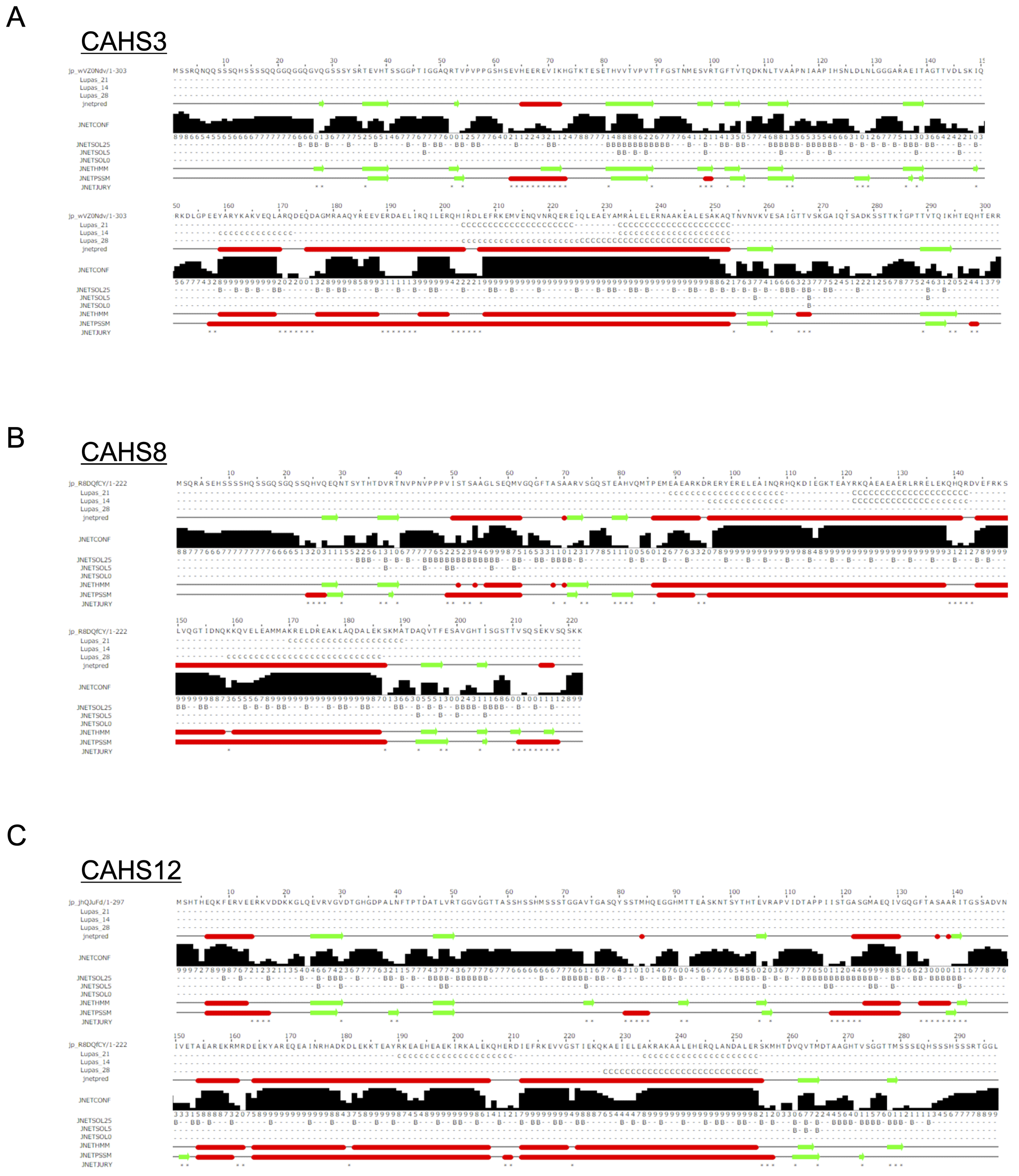

Supplement: S16 Fig — Secondary structure predictions by JPred4 are shown for CAHS3 (A), CAHS8 (B), and CAHS12 (C). Red boxes indicate putative helical regions and green arrows indicate putative beta sheet regions in jnetpred, JNETHSSM and JNETPSSM, respectively. Lupas shows coiled-coil prediction; “C” or “c” indicate putative coiled-coil region and the capital “C” indicates a higher probability. JNETSOL show solvent accessibility. (TIF) [file pbio.3001780.s016.tif]

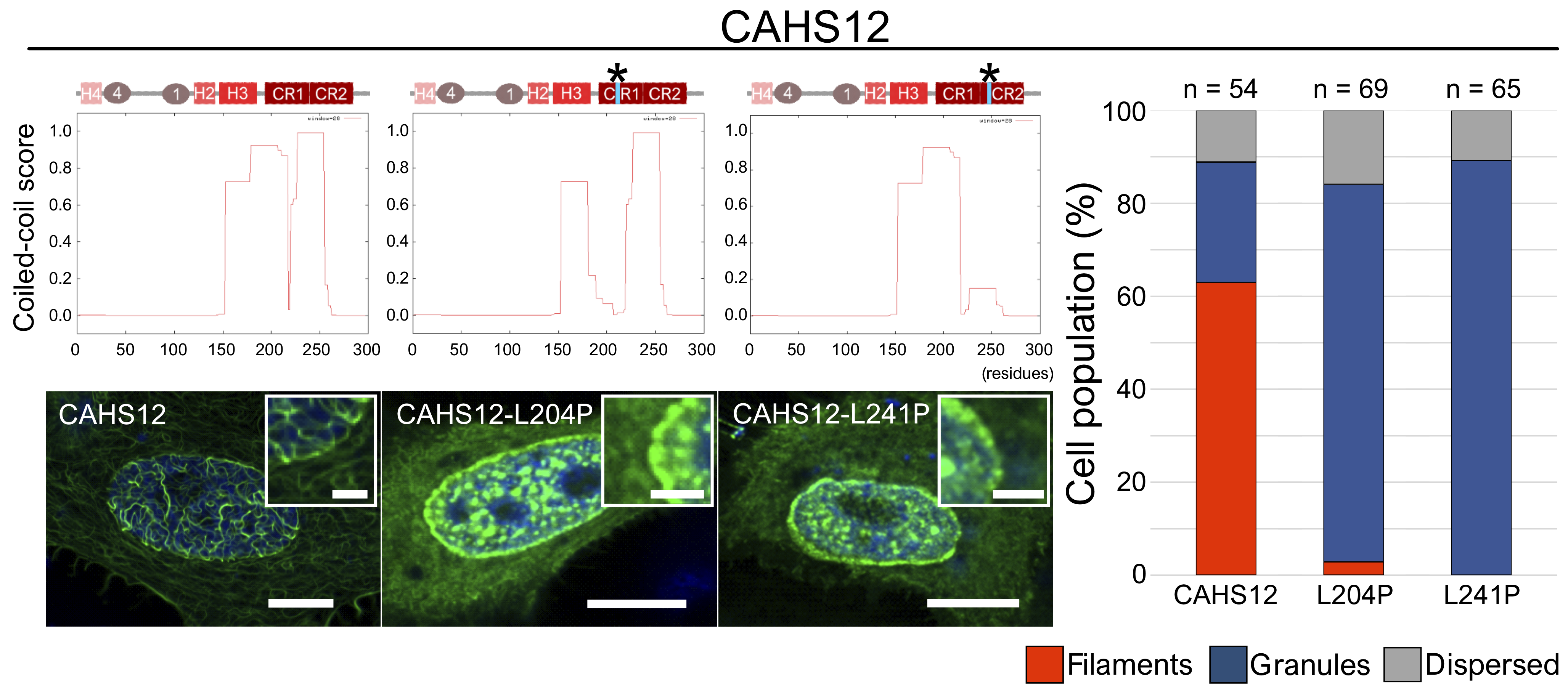

Supplement: S17 Fig — Effects of a helix-disrupting leucine to proline substituting mutation on CAHS12 filament formation are shown. Coiled-coil score predicted by COILS decrease depending on substitution with proline. Asterisks indicate the sites of proline substitutions. Confocal images show representative distribution patterns of the corresponding CAHS proteins (scale bar, 10 μm). Enlarged images are shown as superimposition in each panel (scale bar, 2.5 μm). Blue indicates Hoechst33342 staining of nuclei. The underlying numerical data are available in S4 Data. (TIF) [file pbio.3001780.s017.tif]

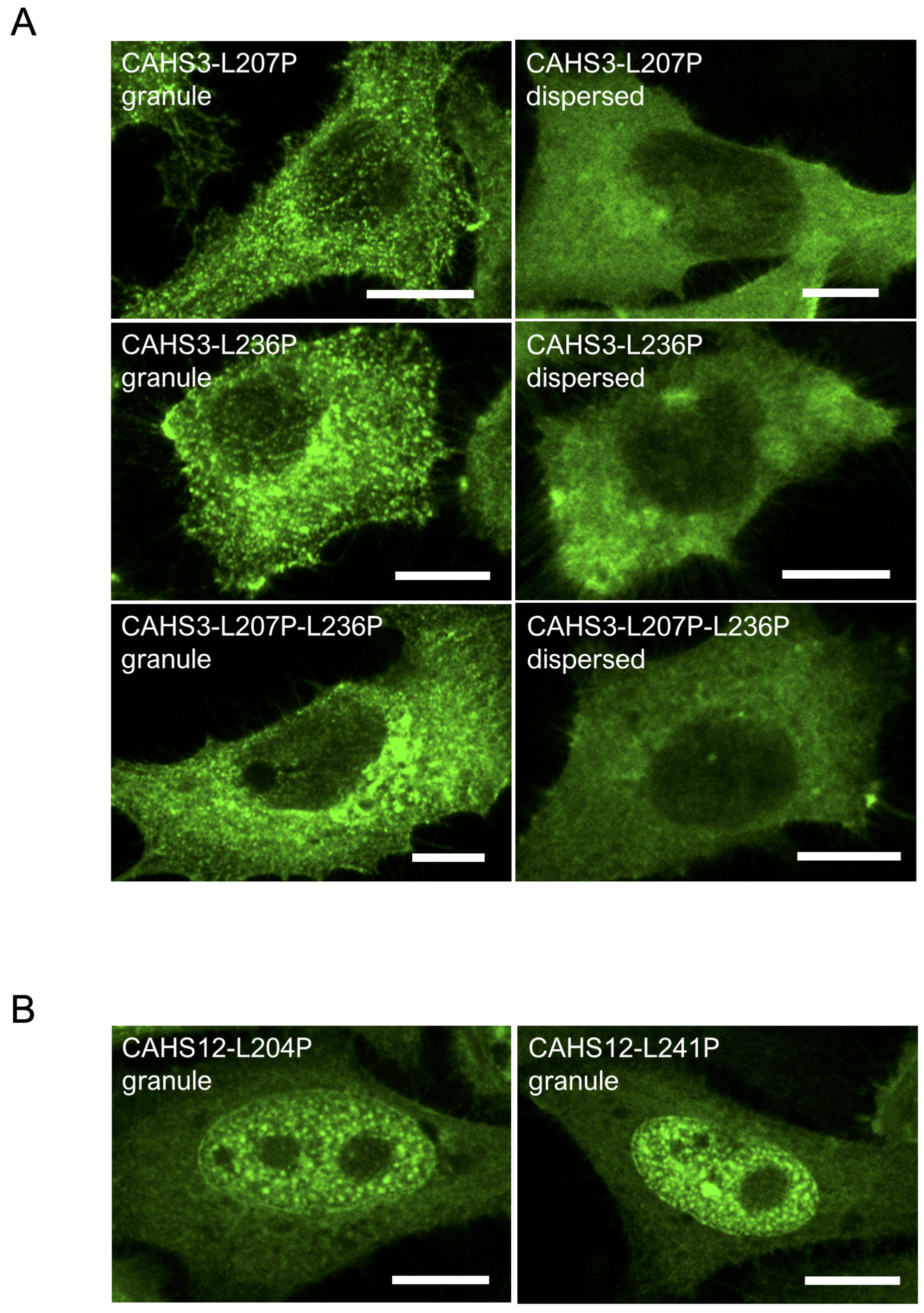

Supplement: S18 Fig — (A–B) Representative images of granule-like condensation or dispersed distribution of proline-substituted mutants of CAHS3 (A) and CAHS12 (B). Scale bar, 10 μm. (TIF) [file pbio.3001780.s018.tif]

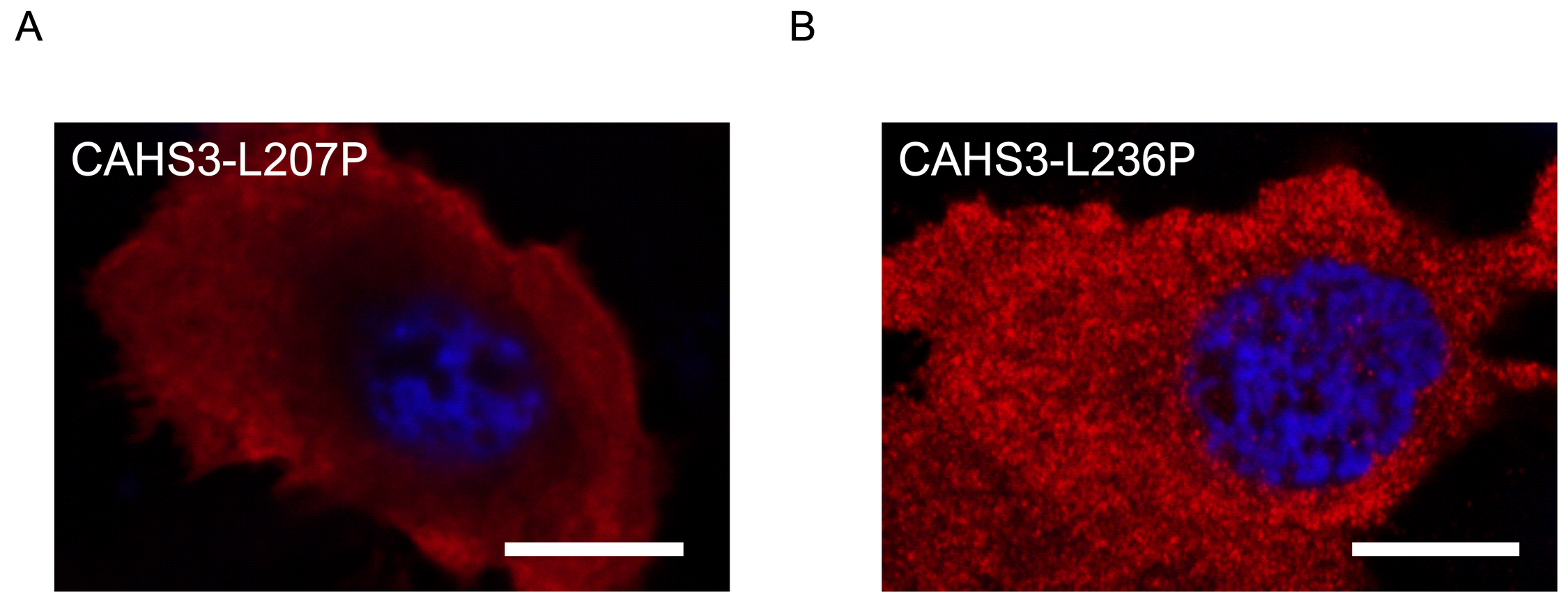

Supplement: S19 Fig — (A–B) Distribution patterns of proline-substituted CAHS3 mutants were examined by immunofluorescence for both CAHS3-L207P (A) and CAHS3-L236P (B). Immunostaining images show the dispersed distribution or slightly condensed granules similar to the corresponding CAHS3 mutants labeled with GFP (Fig 3C). Blue indicates DAPI staining of nuclei. Scale bar, 10 μm. (TIF) [file pbio.3001780.s019.tif]

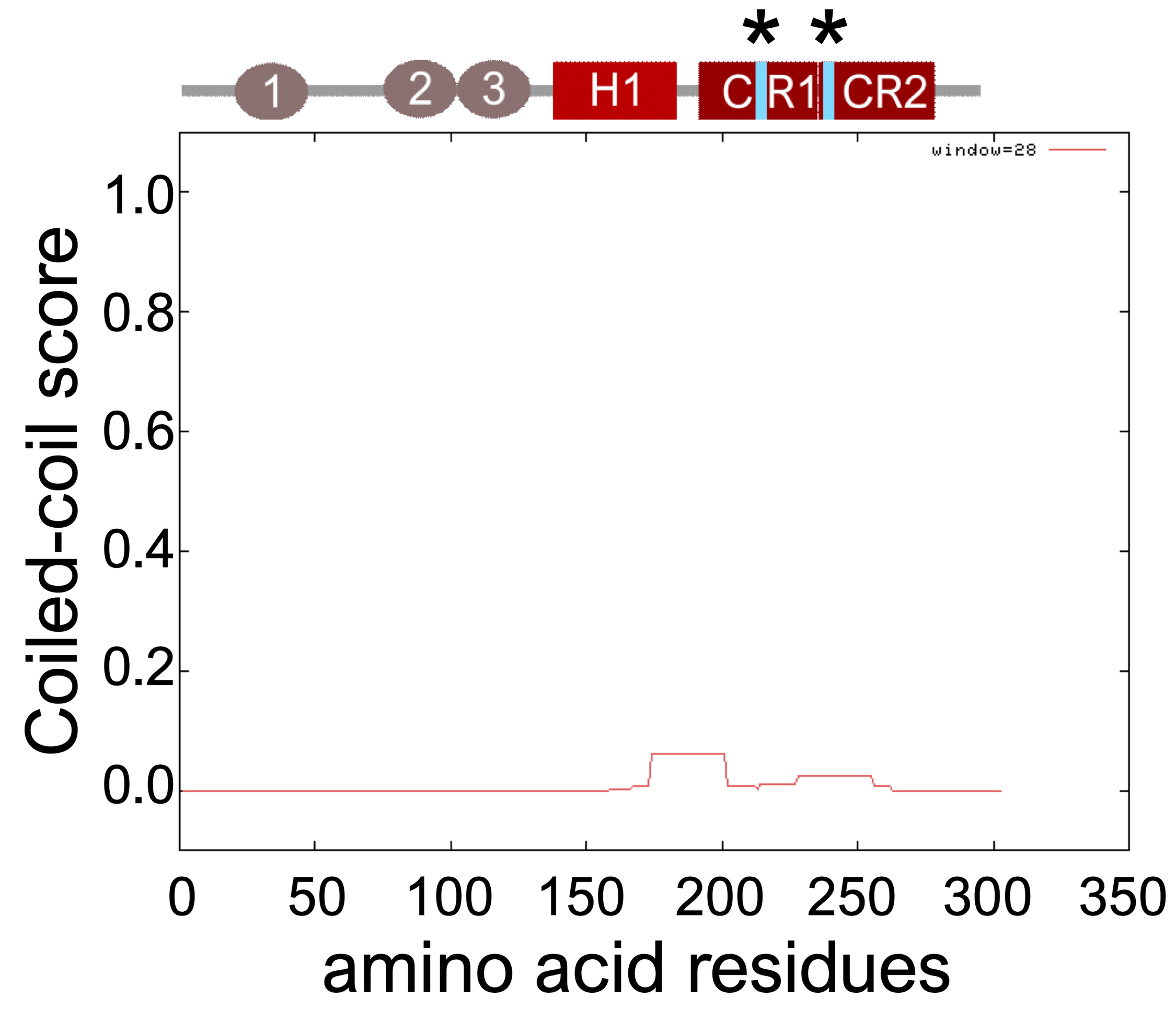

Supplement: S20 Fig — Asterisks indicate the proline-substituted mutation sites. The coiled-coil score was calculated from a CAHS3-L207P-L236P amino acid sequence by a prediction tool, COILS. The underlying numerical data are available in S4 Data. (TIF) [file pbio.3001780.s020.tif]

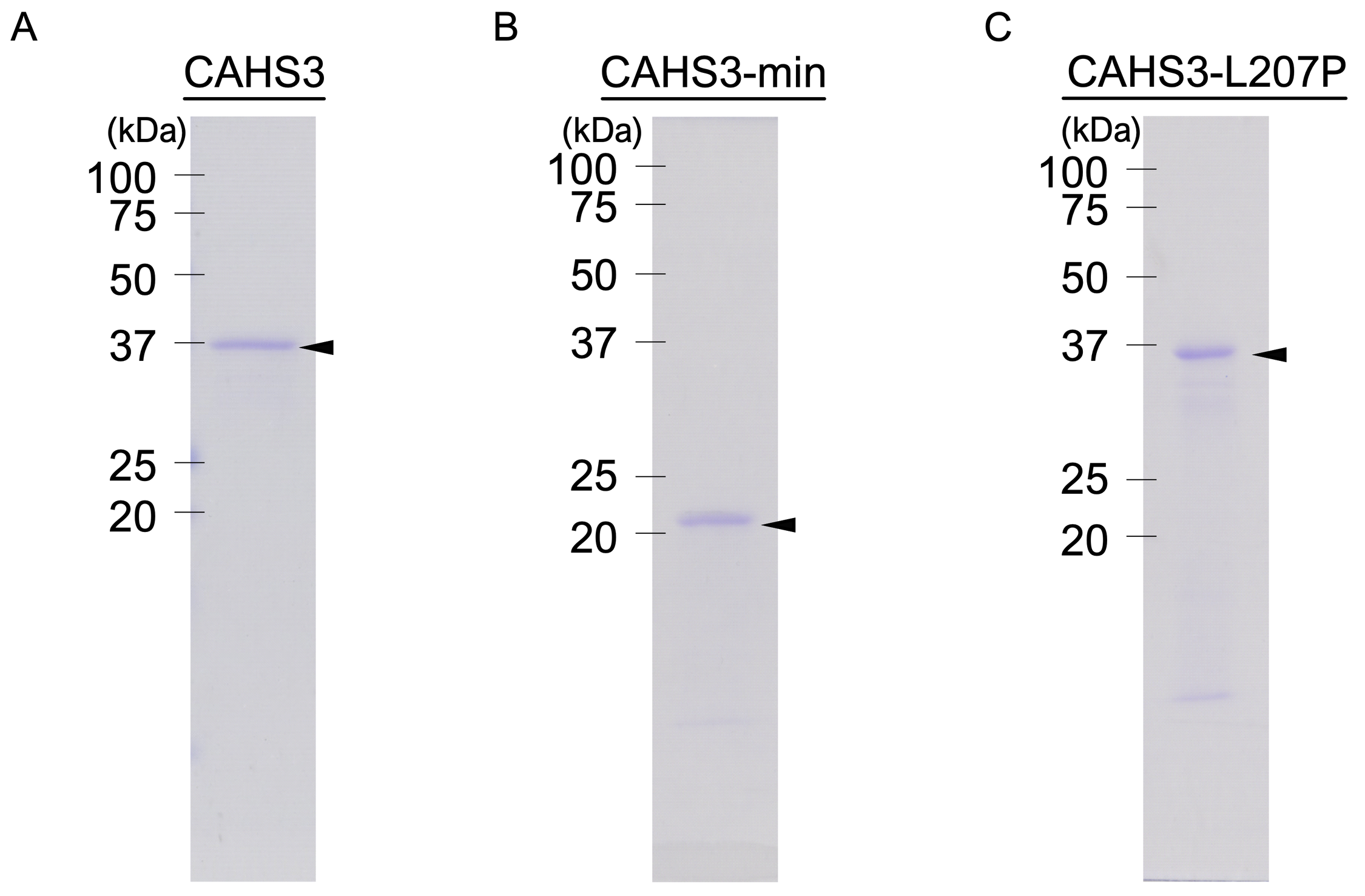

Supplement: S21 Fig — (A–C) Arrowheads indicate major bands corresponding to the expected length of full-length CAHS3 (A), CAHS3-min (B), and CAHS3-L207P proteins (C). (TIF) [file pbio.3001780.s021.tif]

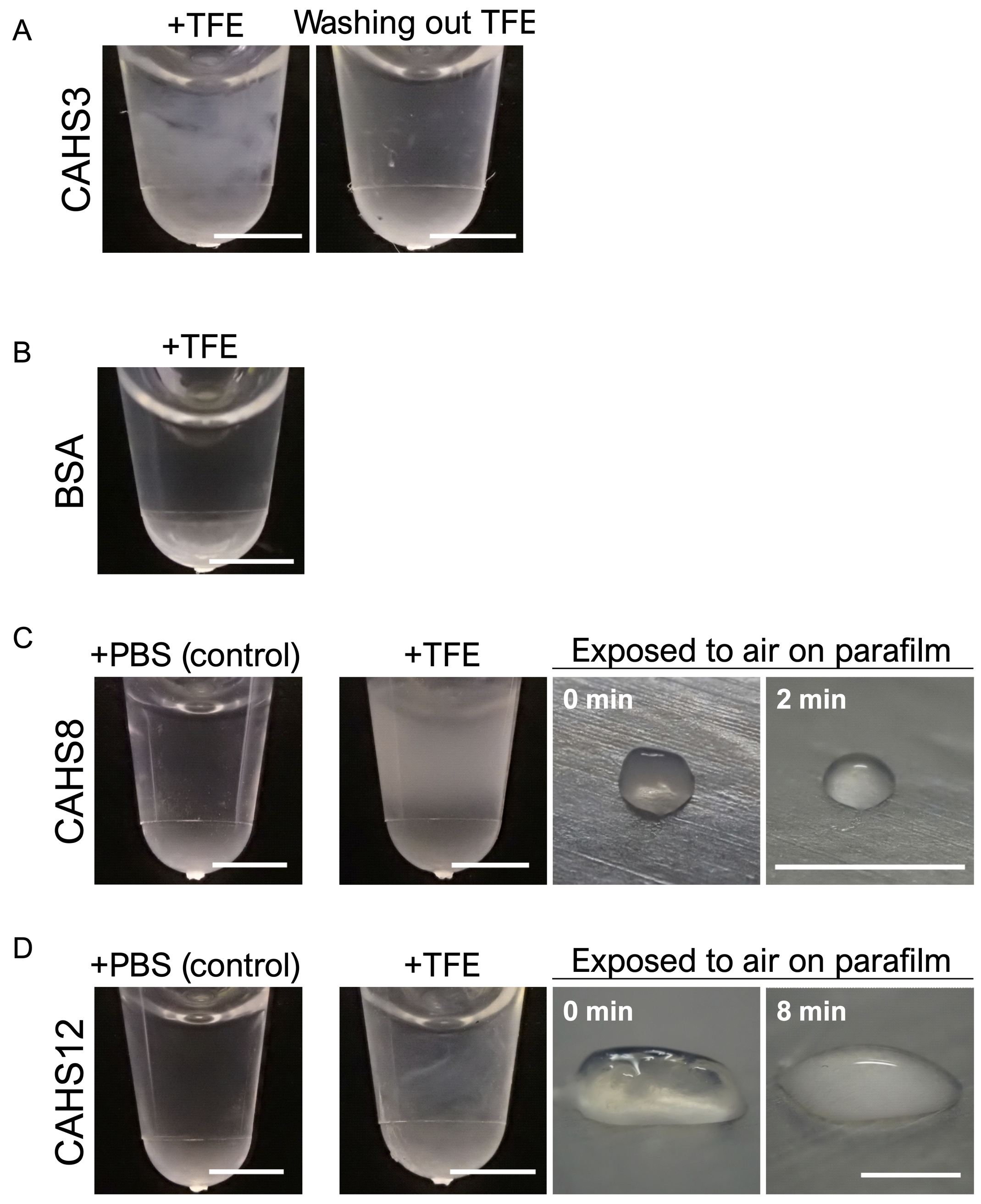

Supplement: S22 Fig — (A) Resolubilization of TFE-dependent CAHS3 gelation. CAHS3 gel condensates induced by TFE (final 20%) were redissolved by rinsing with TFE-free PBS. (B) Effect of TFE on BSA solution. TFE (final 20%) had no visible effect on BSA solution (final 4.0 mg/mL). (C and D) TFE-dependent reversible gelation of CAHS8 and CAHS12 proteins. Addition of TFE (final 20%) caused transient gel-transition of CAHS8 (C) and CAHS12 (D) protein solutions (4.0 mg/mL). These gels spontaneously liquefied within several minutes (shown in white letters) after exposure to air. Scale bar, 2 mm. (TIF) [file pbio.3001780.s022.tif]

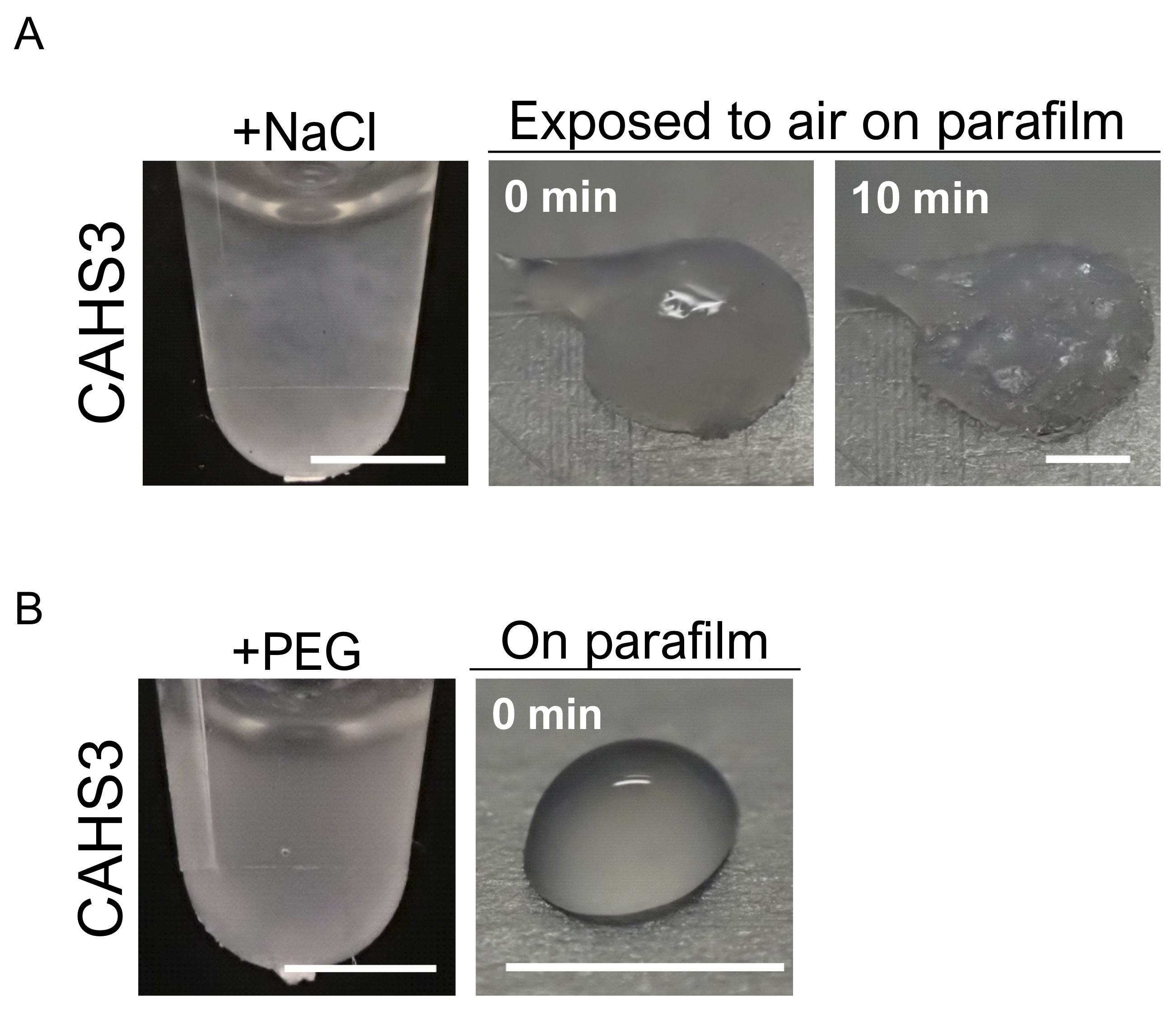

Supplement: S23 Fig — (A) High concentration of NaCl (2 M) caused CAHS3 gelation. The CAHS3 gels induced by NaCl did not liquefy exposed to air for 10 min. (B) Addition of the molecular crowding agent, polyethylene glycol (PEG, final 20%) induced turbidity, but no gelation. (TIF) [file pbio.3001780.s023.tif]

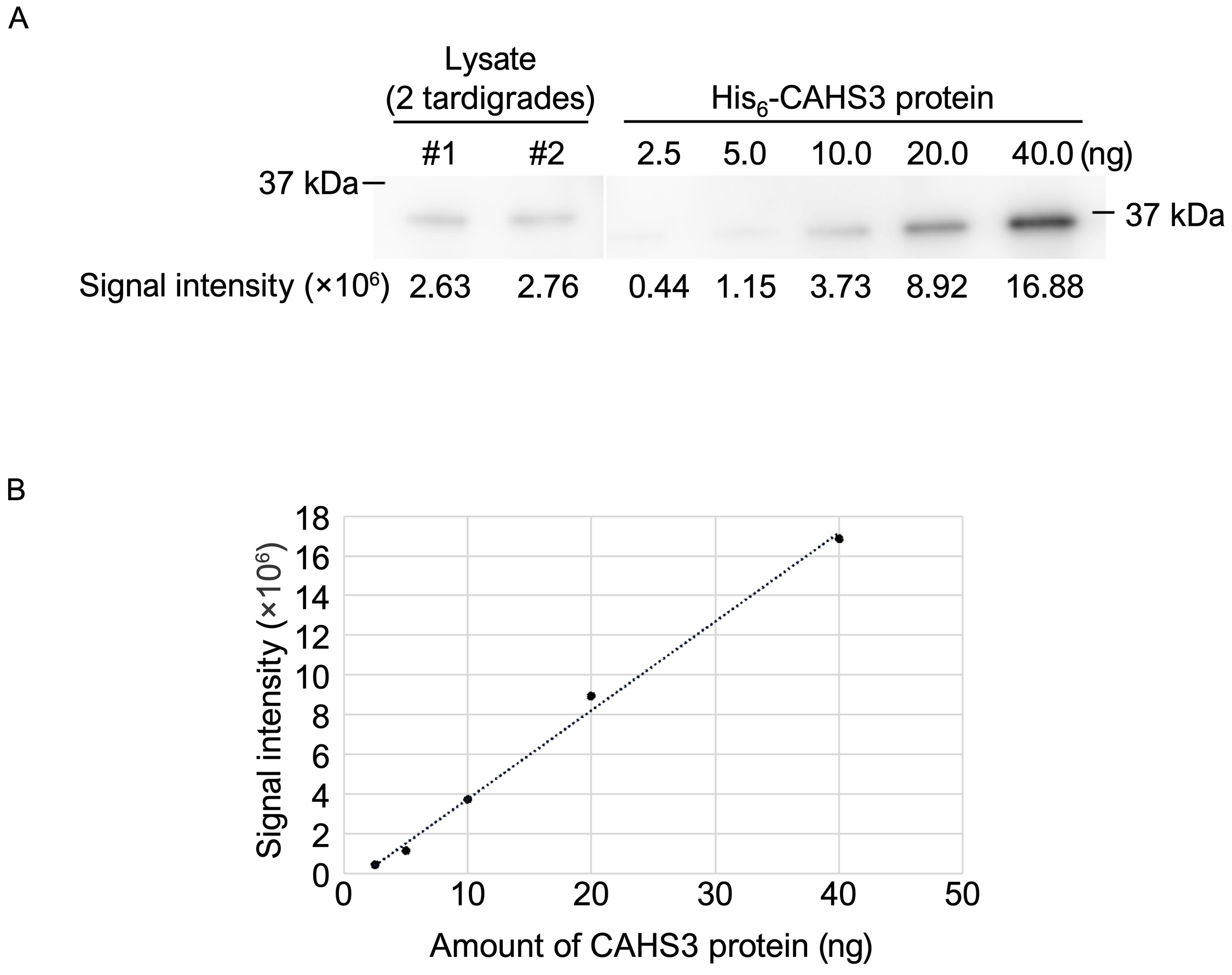

Supplement: S24 Fig — (A) Endogenous CAHS3 protein in R. varieornatus lysate was detected by immunoblotting using anti-CAHS3 antibody. Each lane of the lysate (#1 and #2) contains protein amount corresponding to 2 individuals. Diluted series of recombinant CAHS3 proteins were simultaneously analyzed on the same blot as quantification standards. Due to additional His6-tag, recombinant CAHS3 proteins exhibited slightly higher molecular weight than endogenous ones. Signal intensities were quantified using Fiji imaging software. (B) Based on the immunoblot signal intensity of the diluted series of His6-CAHS3 protein, the standard curve was generated by a linear regression (R2 = 0.9962). The amount of endogenous CAHS3 protein was estimated as about 3.81 ng per tardigrade. The underlying numerical data are available in S4 Data (B). (TIF) [file pbio.3001780.s024.tif]

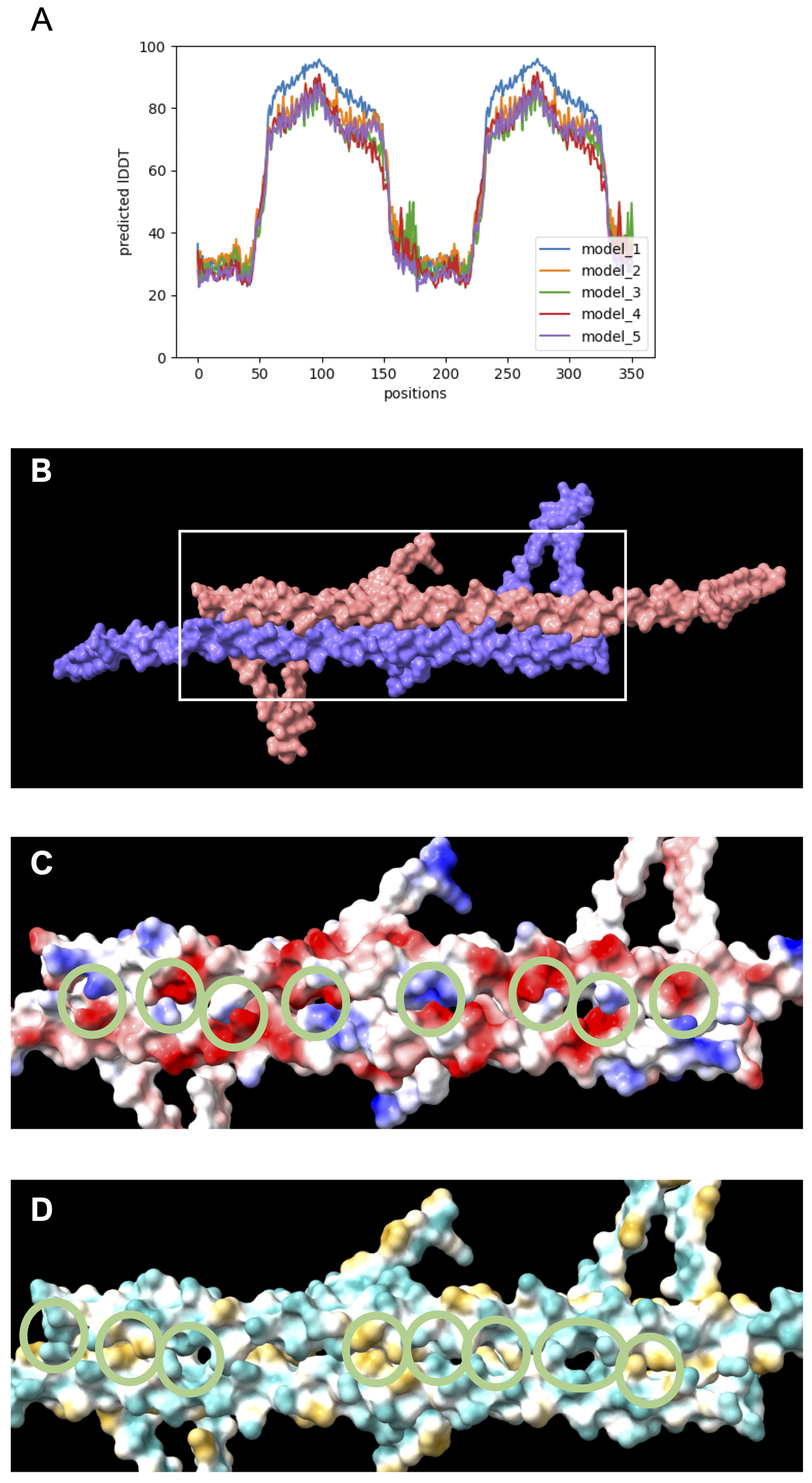

Supplement: S25 Fig — (A) pLDDT scores on the prediction corresponding to a tandem CAHS-min amino acid sequence. Scores corresponding to CR1+CR2 regions (70~90) indicated high structure confidence. (B) Two chains of CAHS3-min proteins distinguished by 2 colors. White box indicates the antiparallel helical region. (C) Magnified view of the charge distributions in the juxtaposed helical regions. Green circles indicate the facing of opposite charges between 2 CAHS3-min proteins, suggesting stabilization by electrostatic interactions. (D) Magnified view of the hydrophobicity distributions. Green circles indicate the juxtaposition of similar hydrophobicities/hydrophilicities between 2 proteins, supporting hydrophobic interactions. The underlying numerical data are available in S4 Data (A). (TIF) [file pbio.3001780.s025.tif]
