# Supplementary material for: Stress-dependent cell stiffening by tardigrade tolerance proteins that reversibly form a filamentous network and gel
Source: PLoS Biol. 2022 Sep 6;20(9):e3001780. doi: 10.1371/journal.pbio.3001780 (PMC9592077; doi:10.1371/journal.pbio.3001780)
Supplement: S1 Table — (PDF) [file pbio.3001780.s026.pdf]

S1 Table Fluorescent-tagged markers for various cytoskeletons and organelles used in this study

Addgene #	Plasmid/Marker name	Target structure	Fluorescent tag
55065	mCherry-keratin-17	Keratin	mCherry
55156	mCherry-vimentin-7	Vimentin	mCherry
85047	mScarlet-I-alpha-tubulin-C1	Tubulin	mScarlet-I
85056	pLifeact-mScarlet-I	Actin	mScarlet-I
98831	mScarlet-I-lamin-b	Lamin	mScarlet-I
85068	pcytERM-mScarlet-I-N1	Endoplasmic reticulum	mScarlet-I
98818	4xmts-mScarlet-I	Mitochondria	mScarlet-I
