# Supplementary material for: Stress-dependent cell stiffening by tardigrade tolerance proteins that reversibly form a filamentous network and gel
Source: PLoS Biol. 2022 Sep 6;20(9):e3001780. doi: 10.1371/journal.pbio.3001780 (PMC9592077; doi:10.1371/journal.pbio.3001780)
Supplement: S1 Raw Images — (PDF) [file pbio.3001780.s034.pdf]

Raw gel image for Fig 1B and S1 Fig

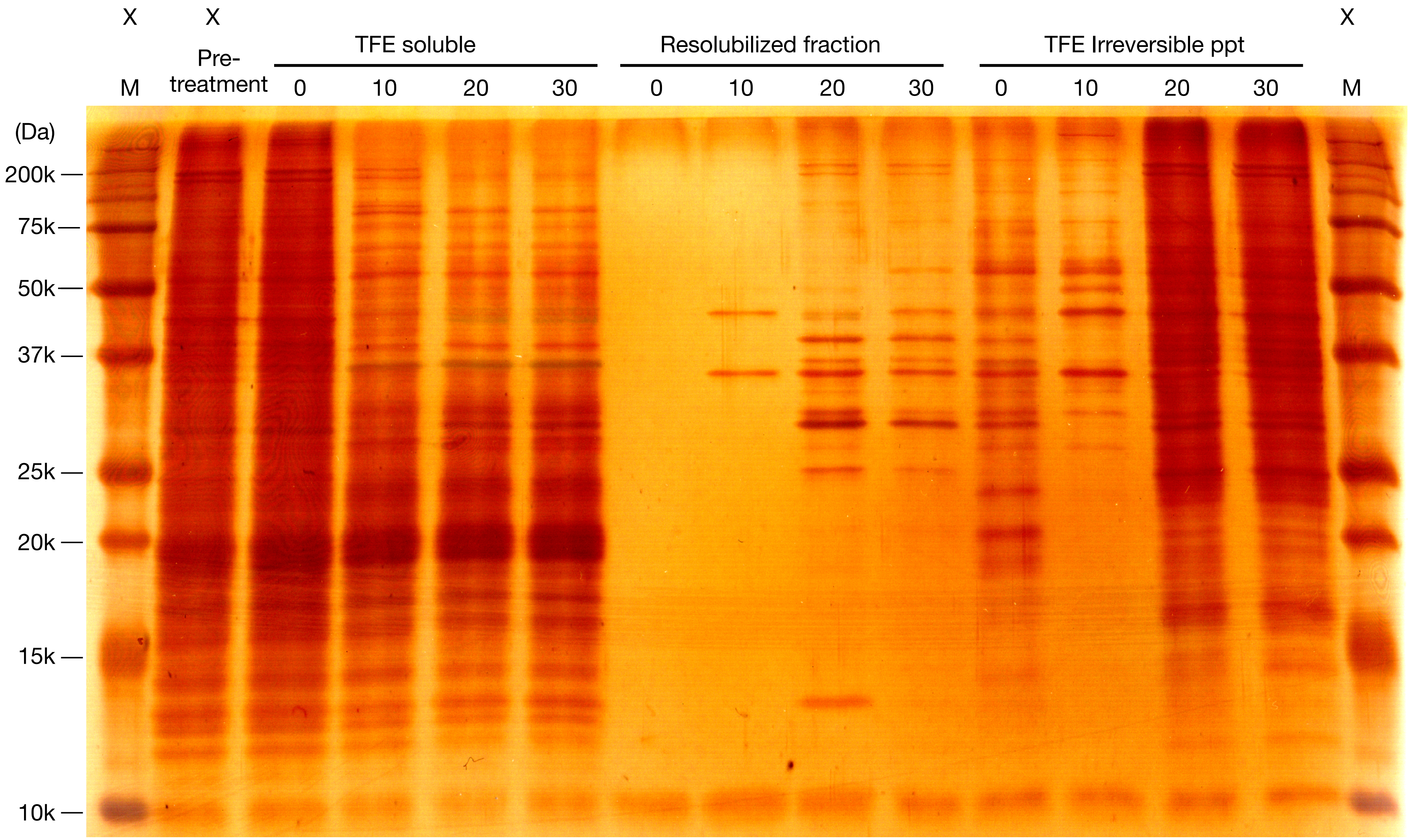

The image of the silver-stained gel was captured with CanoScan LiDE (Canon).

M indicates a molecular weight marker lane: Precision Plus Protein Standards (Bio-rad).

The numbers above lanes indicate the concentrations (%) of treated TFE.

Raw gel image for CAHS3-WT in S21 Fig

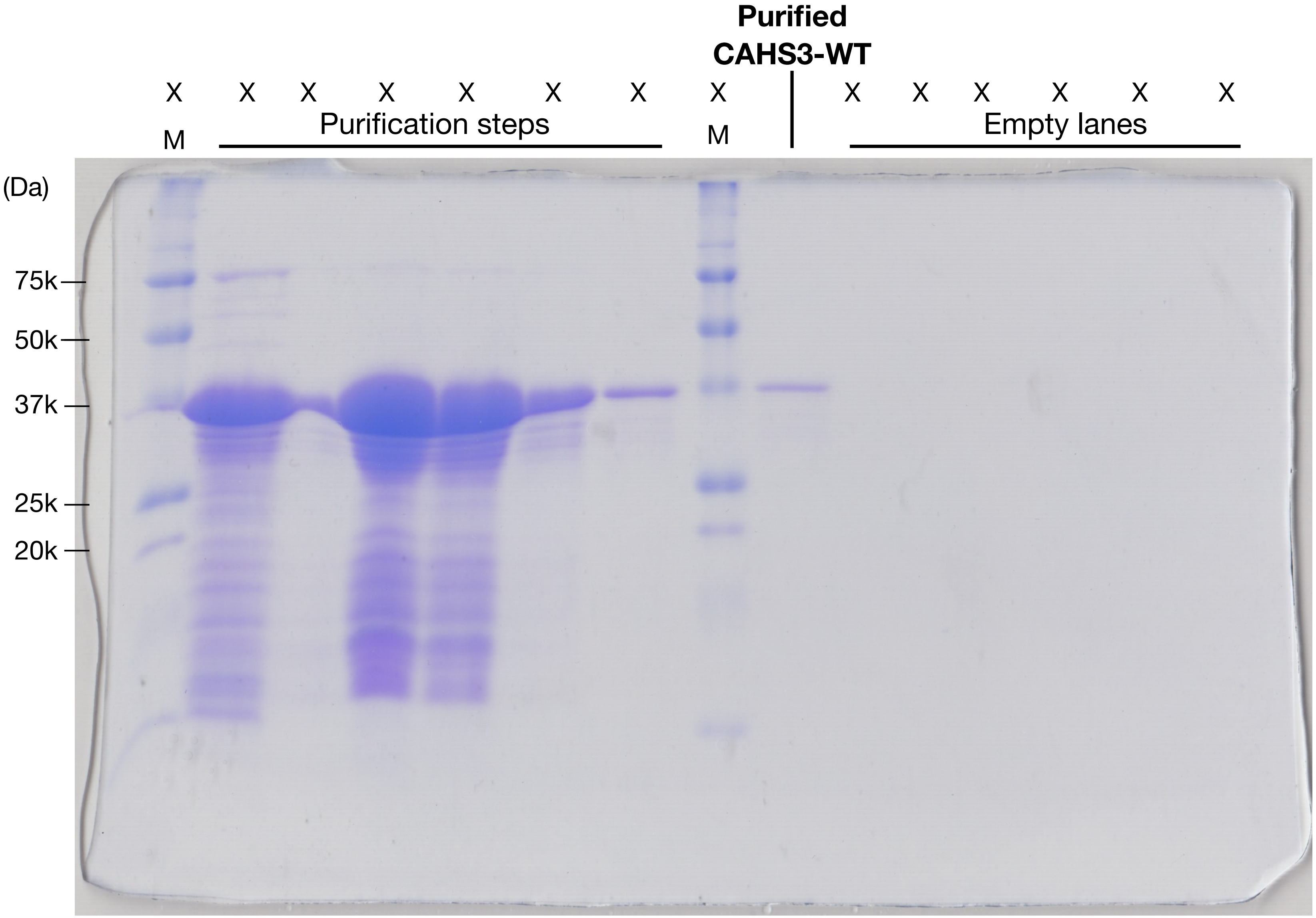

The image of the CBB-stained gel was captured with CanoScan LiDE (Canon).

M indicates a molecular weight marker lane: Precision Plus Protein Standards (Bio-rad).

Raw gel image for CAHS3-min and CAHS3-L207P in S21 Fig

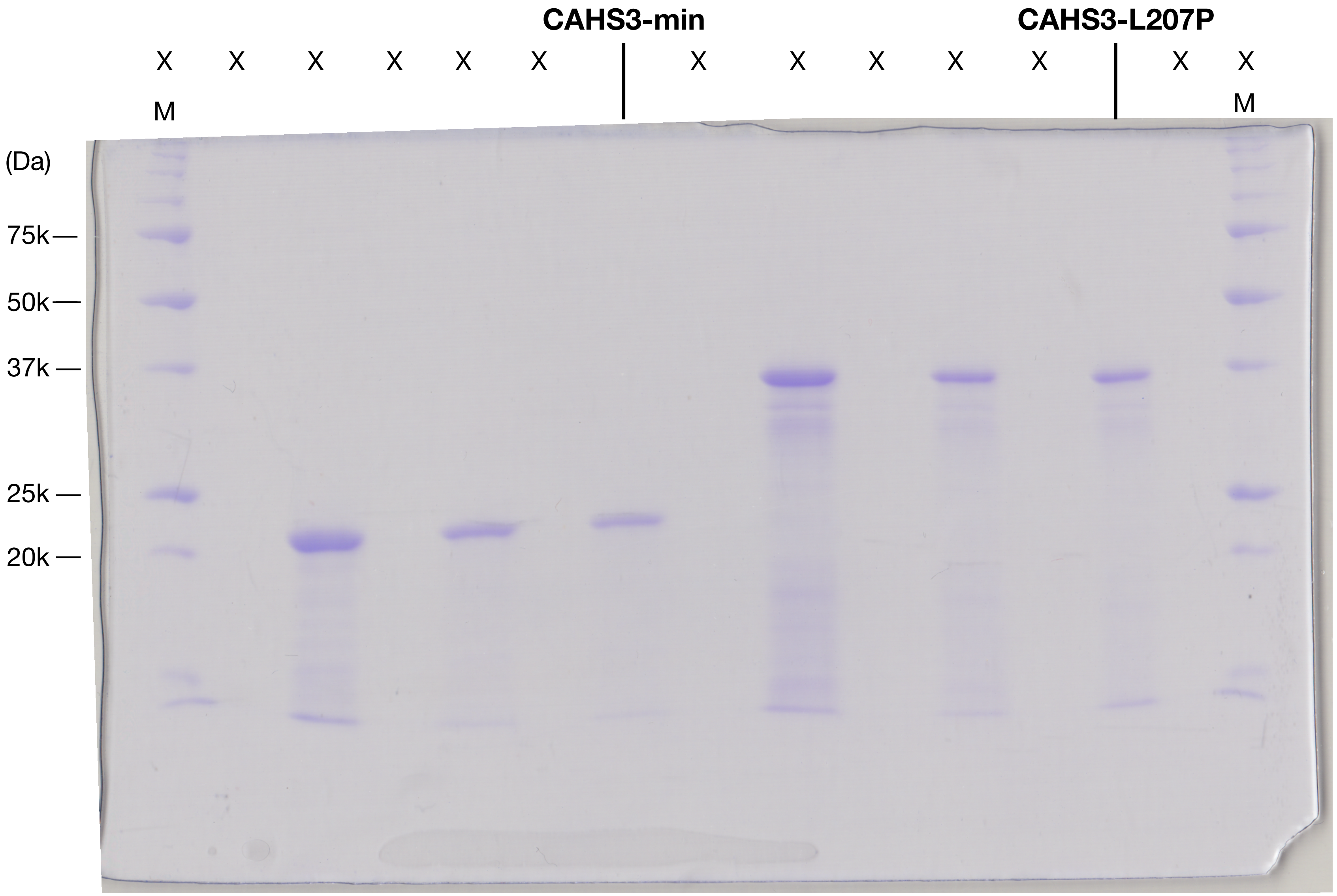

The image of the CBB-stained gel was captured with CanoScan LiDE (Canon).

M indicates a molecular weight marker lane: Precision Plus Protein Standards (Bio-rad).

Raw immunoblotting image for S24 Fig

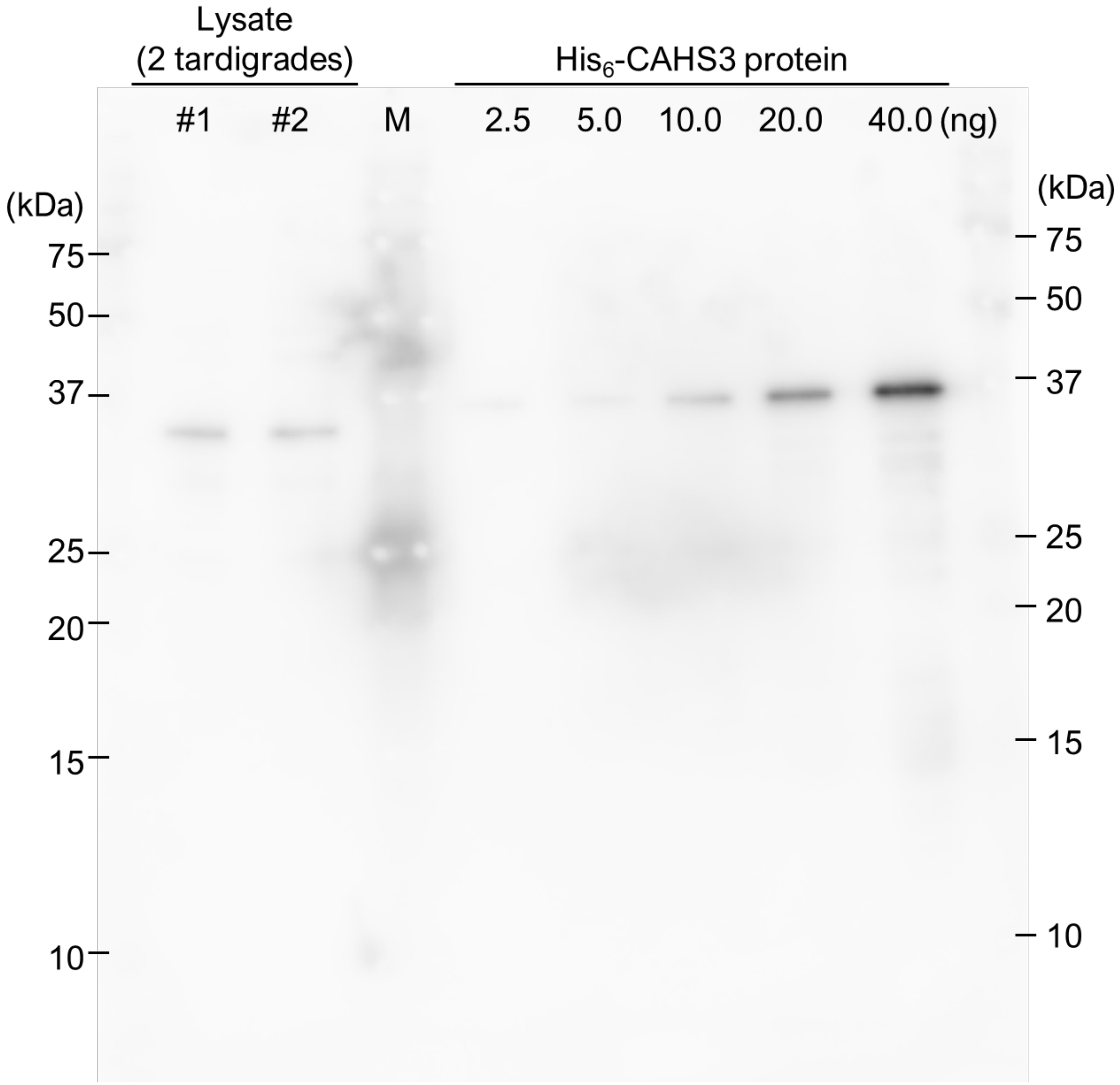

The image of the immunoblotting was captured with ImageQuant LAS500 (Cytiva).

M indicates a molecular weight marker lane: Precision Plus Protein Standards (Bio-rad).
