# Supplementary material for: Stress-dependent cell stiffening by tardigrade tolerance proteins that reversibly form a filamentous network and gel
Source: PLoS Biol. 2022 Sep 6;20(9):e3001780. doi: 10.1371/journal.pbio.3001780 (PMC9592077; doi:10.1371/journal.pbio.3001780)
Supplement: Attachment — Submitted filename: Response_to_reviewers_20220304.pdf [file pbio.3001780.s035.pdf]

We highly appreciated the constructive comments by both reviewers and the editor which greatly helped us to improve our manuscript. Here, we describe our responses to all comments.

NOTE
We updated the sequence and expression data of *Paramacrobiotus* sp. TYO used in Fig 1F-G and part of S1-S3 Data to the published version, because we used slightly different data set in the previous manuscript.

========
Reviewers' comments

Rev. 1:
Tanaka et al. reports reversible stress-induced fiber formation of CAHS proteins, which are tardigrade-unique abundant heat soluble proteins presumed to relate to their anhydrobiotic capabilities. They propose that these set of proteins are novel stress-dependent cytoskeletal proteins. The paper is nicely written, and experimental designs are very comprehensive and thorough, and the beautiful microscope images of filaments in human culture cells are convincing.

I have several comments to possibly improve the paper as detailed below.

1. The authors firstly screen for reversibly soluble proteins using desiccation-mimicking condition with TFE, and name this "Dehydration-dependent reversibly condensing proteins (DRPs)".
Firstly, I would like to suggest a different naming to avoid confusion with DRPs (dynamin-related proteins including Drp1, Dnm1, Dlp1 and so on) that are also frequently involved in stress response. Secondly, I am not certain wether the use of TFE actually mimicks "dehydration". TFE is known as an alpha-inducer, and this may be affecting the type of proteins, and not necessarity dehydration-dependence. As explained thoroughly in the manuscript, CAHS proteins are enriched in helical structures, and other enriched proteins such as actin as well as peroxiredoxins contain large percentage of alpha-helices. I understand that the condition may be too harsh on most proteins, but simply desiccating extracted proteins and rehydrating them could be a more direct protocol to obtain actual "dehydration-dependent reversibly condensing"

proteins, and am curious if the current method with TFE actually recapitulates same set of proteins. This is important, since the authors discuss rather extensively that their method with TFE would see wider potential applications in enriching "dehydration-dependent" proteins.

Thank you very much for your kind and helpful suggestions. We have changed the name of the proteins formerly designated DRPs to DRYPs which stands for "Desolvation-induced ReversiblY condensing Proteins" in response to the reviewer's comment.
The key feature of our method using TFE is that TFE enables separation of condensed proteins from non-condensed proteins by centrifugation as precipitates and supernatants under a stressed condition. We agree that the usage of desiccation instead of TFE would theoretically be better to capture the desiccation-dependent condensing proteins, but in practical, it is impossible to separate the condensed proteins from non-condensed proteins in a dehydrated (no water) condition. If the reviewer means to separate all resoluble proteins upon rehydration after desiccation, in such an experimental scheme, all hydrophilic proteins will be retrieved regardless of stress-dependent condensation. Those proteins are out of the scope of our study and obviously a different (likely much larger) set of proteins will be obtained because they are not screened by stress-dependent CONDENSATION.
TFE is known to induce the conformational changes (largely helix as commented by the reviewer) of several desiccation-tolerance proteins (e.g., LEA proteins or CAHS proteins) similarly as in a dehydrated condition. LEA proteins and CAHS proteins are unrelated in the primary sequence, but both become helix-rich from unstructured state by dehydration. Dehydration could induce conformational change to helix-rich structure of some unstructured proteins and TFE might mimic this conformational change. The identified CAHS proteins exhibited clear reversible condensation (which are predicted from TFE-based screening) in animal cells under a hyperosmotic condition without using TFE, suggesting that TFE-based screening at least partly worked to capture the reversibly condensing proteins under a water-deficient condition. We have added this discussion to the corresponding paragraph in the Discussion section.

2. In the introduction, after stressing the importance of Intermediate Filament (IF), the authors state "Tardigrades have actin filaments and microtubles, but largely lack cytoplasmic IFs, except a tardigrade-unique IF protein called cytotardin". This is a bit

misleading, because the lack of canonical cytoplasmic IFs is a panarthropoda feature. There are many anhydrobiotic arthropods, so please make it clear that this is not tardigrade-unique loss.

Thanks for suggestion. We have described the loss of canonical cytoplasmic IFs as a shared character in Panarthropoda in Introduction. We have also added discussion as other stress-dependent filament-forming proteins might be present in anhydrobiotic arthropods (and heterotardigrades) which also lack the canonical cytoplasmic IFs and our experimental scheme would help to identify such proteins in future, in the Discussion section.

3. Recently, two papers on a very similar topic have been published, and another preprint is available, publication of two of which predates the submission of this paper. Yagi-Utsumi et al. (2021) Sci Rep. reports CAHS1 (R. varieornatus) to reversibly form filaments and gels in vitro, and condensates in human cells, and that the C-terminal region is essential for such fiber formation.

Malki et al. (2021) Angewandte Chemie reports CAHS8 (H. exemplaris) to reversibly form filaments and gels in vitro.

I think the current manuscript still has its own significance, but nevertheless discussions regarding similarities and differences of these closely related works would strengthen the presented arguments.

Thank you very much for evaluation of the significance of our manuscript. As pointed by the reviewer, two related papers were published in mid to late November, during the review process of our manuscript (we submitted this manuscript on 19th Sept as the initial submission). Two papers described the fiber and gel formation of CAHS proteins *in vitro*. One paper describes stress-responsive granule formation in human cultured cells. Both papers suggested CAHS proteins become alpha-helical in gel-transition by elaborate NMR analyses or CD spectrometry, though no causal relationship between helical structure and fiber formation was provided. We have added explanation about these two publications and discussed similarities and differences with our data: Briefly, the enriched helical structure in C-terminal region is in a good agreement with our data; We further provided the evidence that such secondary structure is important for fiber formation through protein mutational analyses. The reported granule formation of CAHS1 in human cells under hyperosmosis is similar to our observation on CAHS8 protein. And we further demonstrated that the granule-forming CAHS proteins could be

integrated to fiber structures of other filament-forming CAHS proteins when co-expressed.

4. The authors use HEp-2 cells in this work. They also use Drosophila S2 cells, so the observed fibrilation is not cell-line specific, but I am curious for the choice of this cell line, because the previous works by the authors' lab consistently utilized HEK293T cells (for example, Yamaguchi et al. 2012, Tanaka et al. 2015, and Hashimoto et al. 2016). It would be interesting to compare the differences if somehow fiber formation was not feasible in HEK293T.

We are glad as you have kindly read our previous publications. Yes, we used HEK293 cells in two previous publications (Yamaguchi *et al*. 2012 and Hashimoto *et al*. 2016), but we used HEp-2 cells in the other previous publication as well (Tanaka *et al*. 2015) in which we examined mitochondrial localization of several proteins. The reason is simple. HEK293 cells are low sticky and easily detach from the dish surface especially in a stressed condition. The detached cells become a round form and are not good for inspection of the precise sub-cellular localization of proteins, because the detached round cells freely move during observation and also provide only a small cytoplasmic area in an optical section. Hep-2 cells are much more sticky and provide better microscopic images in detail. This study and the previous mitochondrial study require a fine resolution and thus we used HEp-2 cells in this study as well. To clarify this, we have added the following sentence in the Methods section: "*We used HEp-2 cells for live-imaging of fluorescently-labeled proteins because HEp-2 cell were well sticky even under a stress condition and enabled precise inspections.*".

5. The authors show fibril formation in Drosophila S2 cells, which is convincing. I think this is a perfect opportunity to test the actual cytoskeletal function of CAHS (if there actually is), since the current paper provides no evidence regarding actual protective role of CAHS upon dehydration or stress-induction. Since Drosophila also lack IFs like tardigrades, was there any phenotypic differences regarding tolerance in S2 cells expressing CAHS? It would really strenghen the argument if the authors can actually show that these proteins "effectively counteract the deformative forces" (Line 104).

Thank you very much for important suggestions about new experiments which actually we had planned to do. We first established the stably transfected S2 cells and performed three additional experiments. 1) We measured the cortical elasticity of the transfected cells and revealed that CAHS3-expressing cells exhibited significantly higher elasticity than the control cells under a hyperosmotic condition. 2) We also measured the change of cell volumes upon hyperosmotic stress and showed that CAHS3-expressing cells exhibited less shrinkage (i.e., better retention of cell volume) under hyperosmosis. 3) Finally, we examined the effect of CAHS3 on cell integrity under hyperosmosis using PI-exclusion assay and demonstrated that CAHS3-expressing cells exhibited better survival against hyperosmotic stress. As noted, all of the examined experiments supported our notion. We have put these new data in a new figure (Fig 9) and described in a new sub-section. We believe that our additional data satisfactorily solve the concern raised by the reviewer.

Minor comments:
Proteomic analysis data could be deposited to one of ProteomExchange repositories.

According to the reviewer's suggestion, we have deposited the mass spectrometry proteomics data to the ProteomeXchange Consortium via the jPOST repository with the dataset identifier PXD030241. We have described this deposition with the accession number in the corresponding Materials and Methods section.

Rev. 2:
This manuscript deals with the putative function of the newly discovered CAHS proteins, which belong to the so-called "tardigrade unique proteins" found exclusively among eutardigrades. The manuscript is well-written and based on thoroughly conducted experiments. Please find my more detailed comments below.

Conclusions are generally supported by the data. My only major concern is that you over interpret your data, when you neglect to mention that CAHS proteins are missing in one of the two major evolutionary lineages within the tardigrades. In other words, as CAHS proteins are found exclusively among eutardigrades, they may not be as

important for anhydrobiosis as perceived from reading your manuscript. Consequently, I recommend that you briefly discuss papers showing that anhydrobiotic heterotardigrades lack CAHS proteins (Kamilari et al. 2019. BMC Genomics 20, 607; Murai et al. 2021. BMC Genomics 22, 813) and that you avoid statements such as "....(CAHS) proteins which are essential for the anhydrobiotic survival of tardigrades". The latter and other comparable statements are simply not in accord with current knowledge within the field. Along this line, I suggest that you in the title and throughout the abstract, introduction etc. replace the word "tardigrade" with the more accurate term "eutardigrade".

We agree with the reviewer's comments. We have added the detailed explanation in Introduction to avoid a misunderstanding and have changed the words and phrases accordingly in the manuscript. One exception is the title because the journal is intended to be open to broad readers and we think that the word 'eutardigrade(s)' is unfamiliar and too technical for most readers to understand the title before reading the content. As described above, we added the detailed explanation about the limited presence of CAHS proteins in eutardigrades in Introduction (and concisely in Abstract) which keeps readers satisfactorily apart from misunderstanding after reading the manuscript.

You show that cytoskeletal proteins, such as actin, are significantly enriched in DRPs. I lack a comment on the possible significance of well-known cytoskeletal proteins in relation to stabilization of tardigrade cell structure during desiccation.

We have added discussions about the possible mechanism and role of the enriched proteins in DRYPs including cytoskeletal proteins and translational machinery as "In the DRYPs, stress-related unstructured proteins including CAHS and LEA proteins were enriched (Fig 1E and G), as well as translational proteins and cytoskeleton-related proteins (Fig 1D). These proteins might be incorporated into stress-dependent condensates like stress granules to be protected from stress. Alternatively, some of them like cytoskeletal proteins might be co-precipitated through entangling with CAHS filaments."

In the introduction (and discussion) you briefly discuss and dismiss the possibility that CAHS proteins contribute to vitrification of the cytoplasm during desiccation. It would

be interesting if you could present a more elaborate comparison between vitrification and the gel-transition observed in the current manuscript. What happens to the CAHS hydrogels, if exposed to very low relative humidity (and extremely low temperatures or pressures)?

In this manuscript, our main focus is on identification and analysis of stress-dependent condensing proteins e.g., CAHS, not on denying the vitrification hypothesis and thus we did not examine the possibility of the vitrification in detail. Therefore, we think it is not an adequate place to present an elaborate comparison with vitrification and rather we would like to comment that our observation is more consistent with the water retention in gel structure. During the review process, the gel transition of other CAHS proteins has been reported in two other recent publications and thus we do not think the elaborate comparison with vitrification is needed in this manuscript. The effect of temperatures on CAHS gels was reported in one of these two publications.

You state that "In vitro gel transition was observed when using a relatively high concentration (~ 4 mg/mL) of CAHS solution". The manuscript would benefit from a discussion on whether the used concentrations of CAHS proteins are physiological relevant. What are the presumed concentrations of the proteins within cells of Ramazzottius varieornatus in the active and desiccated states?

Thanks for the suggestion. We performed the rough quantification of endogenous CAHS3 proteins in tardigrade by immunoblot analysis. The amount of CAHS3 proteins were calculated as about 3.8 ng per individual. The wet weight of an individual of *R. varieornatus* is about 1.84 µg and thus the concentration of CAHS3 protein is estimated roughly 2 mg/mL. Considering that CAHS3 proteins are present only in the cytosol, the physiological concentration should be much higher than the estimated value and we assumed that it is not far from the concentration used for gel-transition experiment. We have added this estimation and the corresponding discussion in the Discussion section. The data is presented in a new S20 Fig.

Please carefully check figures and text for minor errors. E.g. correct to "Relative" on y-axis in Fig 2C and explain white arrowheads in Fig 2F. The images of S2 cells presented in S8 Fig seem to be of low quality.

Thanks for pointing out errors. We carefully checked the manuscript thoroughly and corrected the found errors. As for the images of S2 cells, it is difficult to capture the images at the quality similar to those of human cultured cells, because S2 cells are much smaller than human cells. To acquire the better images, we recaptured the images using Zeiss confocal microscopy with AiryScan2 which offers better resolution, and replaced the images of S2 cells in S8 Fig.
