# Supplementary material for: Stress-dependent cell stiffening by tardigrade tolerance proteins that reversibly form a filamentous network and gel
Source: PLoS Biol. 2022 Sep 6;20(9):e3001780. doi: 10.1371/journal.pbio.3001780 (PMC9592077; doi:10.1371/journal.pbio.3001780)
Supplement: Attachment — Submitted filename: Response_to_reviewers_20220602.pdf [file pbio.3001780.s036.pdf]

Here, we describe our responses to all reviewer's comments.

NOTE

According to the editor's recommendation, we have reduced the number of main figures of our manuscript and accordingly have reorganized the subsections in Results section. We also updated the species name of *Paramacrobiotus* sp. TYO to *Paramacrobiotus metropolitanus* as described in the recent publication (Sugiura *et al*., Zootaxa 2022).

========

Reviewers' comments

Rev. 1:

The authors have put an extensive effort in revising the manuscript, and the addition of new experiment using S2 cells is fantastic in showing that the expression of CAHS3 in heterologous condition confers osmotic stress tolerance. Induction or enhancement of desiccation tolerance in heterologous cells has been reported by Boothby et al. (2017) Mol Cell, but the survival rate was extremely low in the above work, so a more definitive confirmation in a host more closely related to tardigrades is an important progress.

Thank you very much for your kind and encouraging comments.

I appreciate the consideration in changing the name of DRYPs to avoid confusion. The authors, however, seems to misunderstand the fact regarding the alpha-inducing property of TFE. The authors state in the cover letter "TFE is known to induce the conformational changes (largely helix as commented by the reviewer) of several desiccation-tolerance proteins (e.g., LEA proteins or CAHS proteins) similarly as in a dehydrated condition." and "Dehydration could induce conformational change to helix-rich structure of some unstructured proteins and TFE might mimic this conformational change." But in fact TFE is a well-known alpha-inducer commonly used in protein engineering, not specific to "desiccation-tolerance proteins", which stablizes the alhpa-helical structure but not the beta-region (there are numerous papers, but see for example, Shiraki et al. 1995 JMB). My original concern (which remains to be addressed) is that "Desolvation-induced ReversiblY condensing Proteins (DRYPs)" proposed may actually be "Alpha-induction-based Reversibly condensing proteins" and may not necessarily mimic desiccation. I understand, and the data in Fig.1 nicely shows, that there is an overlap - CAHS and LEA happen to be good alpha-formers and get enriched - but this may be a partial picture of

desiccation treatment, and there is still little evidence that desiccation itself actually promotes alpha-helical structures universally in the proteins. Therefore, while I understand the potential of this method, I am not fully convinced for the validity to call it "Desolvation-...", and even if so, limitation of this method should be discussed, that it may significantly overlook structural changes other than those in alpha-helix, and that there could be artefacts related to the alpha-inducing nature of TFE (which might not necessarily happen upon desiccation universally in all proteins).

Thank you very much for your thoughtful comments. We agree that TFE is frequently used as a stabilizer of the alpha-helical structures of protein. Several modes of action have been proposed for the mechanism of helix-stabilization by TFE, but these can be categorized mainly to two-groups: 1) the displacement of water molecules from the surface of polypeptides or 2) the destabilization of (= increase of the energetic/entropic/enthalpic costs in) the interaction between polypeptide and solvent (water molecules), both of which indirectly promote intramolecular hydrogen bonding in polypeptides and stabilize the secondary structures of proteins. The stabilization effect on the intramolecular hydrogen bonding by TFE is stronger in local (among near residues) and thereby alpha-helical structure is more effectively stabilized, though beta-structures are also stabilized in infrequent cases (cf., the recent review, Vincenzi M *et al*., Curr Protein & Peptide Science, 2019). We also agree that TFE-induced conformational change would somewhat overlap with but not perfectly match with the dehydration-induced change for all proteins as commented by the reviewer. Therefore, to clarify that our method is based on the usage of TFE not perfectly mimicking dehydration/desolvation, we have further changed the name of the isolated proteins from "Desolvation-induced reversibly …." to "TFE-dependent reversely …". This change clarifies the limitation of our method as suggested by the reviewer.

We have described the explanation about the link between alpha-inducing activity and the desolvation property of TFE on polypeptides with the caveats on the possible side-effect by alpha-inducing property in our method in the corresponding part of Discussions, as "*TFE is also known as a stabilizer of helical structure[45], for which several stabilization mechanisms have been proposed; e.g., the destabilization of the interaction between polypeptides and water molecules promotes local intramolecular hydrogen bonding in polypeptides and consequently stabilize the helical structure [27,28,46]. Our T-DRYPs isolation method could capture CAHS and LEA proteins through TFE-induced helix formation as occurred in a dehydrating condition. We also cannot exclude the possibility that some proteins in T-DRYPs could be isolated through*

the helix-inducing property rather than the desolvating property of TFE." (p.20, ls. 511-517).

We also cited the corresponding literatures including one suggested by the reviewer as follows.

27. Roccatano D, Colombo G, Fioroni M, Mark AE. Mechanism by which 2,2,2-trifluoroethanol/water mixtures stabilize secondary-structure formation in peptides: a molecular dynamics study. Proc Natl Acad Sci U S A. 2002;99: 12179-12184. doi:10.1073/pnas.182199699

28. Kentsis A, Sosnick TR. Trifluoroethanol promotes helix formation by destabilizing backbone exposure: desolvation rather than native hydrogen bonding defines the kinetic pathway of dimeric coiled coil folding. Biochemistry. 1998;37: 14613-22. doi: 10.1021/bi981641y

45. Shiraki K, Nishikawa K, Goto Y. Trifluoroethanol-induced stabilization of the alpha-helical structure of beta-lactoglobulin: implication for non-hierarchical protein folding. J Mol Biol. 1995;245: 180-194. Doi: 10.1006/jmbi.1994.0015

46. Vincenzi M, Mercurio FA, Leone M. About TFE: Old and new findings. Curr Protein Pept Sci. 2019;20: 425-451. Doi: 10.2174/1389203720666190214152439

Again I congratulate the authors on the additional work in Fig.9 regarding the osmotic stress tolerance of S2 cells. It nicely adds a convincing evidence that these heat-soluble proteins actually contribute to desiccation tolerance in cells. I, however, still am not fully convinced that the CAHS3/12 fibers are "cytoskeletal", since the control experiment is only an empty vector and not non-fiber producing CAHS (like CAHS8 or truncated CAHS3 or 12). Increase in osmotic stress tolerance in non-fiber producing LEA-family proteins are reported elsewhere (Hibshman and Goldstein 2021 BMC Biology, or the authors' own work Tanaka et al. 2015 PLoS One), so in order to confirm that the filamentation is essential and "cytoskeletal", it requires comparison to non-"cytoskeletal" gels and significant higher tolerance over them.
Yet again, I think the current work nicely demonstrates with beautiful microscopy that these proteins actually form filaments in cells, and I find the discussion and speculation that these proteins serve putative functions like cytoskeletons inspiring. However, I still do not think there is strong evidence for the cytoskeletal functionality, to include and signify it in the title.

Thank you very much for a high evaluation of our S2 cell experiments and careful consideration. We agree that the improved osmotolerance alone did not fully prove the cytoskeletal functionality of CAHS proteins. We also understand the reviewer's concern to suggest the mutant analysis and thereby have explicitly described the caveats on this point as "*but it is not ascertained whether this enhancement of tolerance fully depends*

upon the filament formation by CAHS3. CAHS3 might contribute to the tolerance in an alternative way, and our model does not also exclude other possible contributions or functionality of CAHS proteins." in the corresponding part in Discussion section (p.18, ls.452-455).

We have also toned down the claim on the cytoskeletal functionality throughout the manuscript including the title; We have simply described the formation of cytoskeleton-like filamentous network in Results section and then discussed its potential functionality in Discussion section. The new title is "*Stress-dependent cell stiffening by tardigrade tolerance proteins* ***reversibly forming*** *cytoskeleton-like filamentous network and gel*".

The osmotolerance experiments using non-fiber CAHS mutant will support the dependence of the tolerance on fiber-formation, whereas whether the CAHS proteins act as a cytoskeleton is another point. Thus, we prefer directly measure/examine the cytoskeletal features provided by CAHS proteins and had provided the data showing the increase of cellular elasticity by CAHS proteins depending on the fiber-forming condition and the suppression of cell shrinkage (the retention of cell volume) against hyperosmosis by CAHS protein; both of which are characteristic features provided by cytoskeletons. We have added the explanation about the meanings of these experiments in Discussion section in more detail to help the readers understood.

Minor comment:

pg.20 Line 506 "although the necessity of such helix structure for filament/gel formation was not demonstrated. " While I agree that the analysis is not as detailed, I think Yagi-Utsumi et al. (2021) Figure S1 rather clearly show the contribution of the conserved helical C-terminal half is essential in filament/gel formation both by NMR and AFM analyses.

We apologize the ambiguous expression used in the previous manuscript. We have changed the expression to be more strict as "*although the effect of disturbance of such helix structure on filament/gel formation had not been examined.*" (p.19, ls.476-477).
