# Supplementary material for: Stress-dependent cell stiffening by tardigrade tolerance proteins that reversibly form a filamentous network and gel
Source: PLoS Biol. 2022 Sep 6;20(9):e3001780. doi: 10.1371/journal.pbio.3001780 (PMC9592077; doi:10.1371/journal.pbio.3001780)
Supplement: Attachment — Submitted filename: response_to_requests20220713.pdf [file pbio.3001780.s037.pdf]

Here, we described our responses to all editorial requests.

========

Editorial Requests

Based on our Academic Editor's assessment of your revision, we are likely to accept this manuscript for publication, provided you satisfactorily address the following data and other policy-related requests (see below).

In addition, we would like you to have a careful look again through the manuscript and make sure that the data are correctly presented (specially the statistics) and referred to in the text. Given the extensive reorganization of the figures and the large number of supplemental figures, it is easy for mistakes/omissions to occur.

We have carefully checked our manuscript thoroughly and mended them where needed. We confirmed that the data are correctly presented and referred to in the text.

We would also like you to consider a suggestion to improve the title:

"Tardigrade cytoskeleton-like proteins can reversibly form a filamentous network and undergo gel-transition in response to stress"

Thank you so much for suggesting the new title and giving us an opportunity to reconsider the article title. The CAHS proteins which are focused in our manuscript have been known as tardigrade tolerance proteins, but have never been proposed as cytoskeleton(-like) proteins. Rather, we proposed the similarity to cytoskeletal proteins based on our data including the filament formation, gel transition and the increase in elasticity shown in our manuscript. Therefore, we are afraid that the logic in the proposed title could be something reversed from the history or the experimental data. As you may know, we had claimed the putative cytoskeleton-like functionality or properties of CAHS proteins based on our data, but we understand there could be objections against such claims as commented by the reviewer. We suppose that the phrase 'cytoskeleton-like' could sound not a solid claim which might be supported only indirectly from the evidence. Of course, we prefer the title directly supported by the experimental evidence as much as possible, and thus we agree to remove the phrase 'cytoskeleton-like' from the title. We have provided the following evidence in our manuscript, though the causal relationships among them are left for future work.

1) CAHS proteins form filamentous network in animal cells in response to stress.
2) CAHS proteins undergo gel-transition *in vitro*.
3) CAHS proteins increase the cell stiffness in response to stress.
4) CAHS proteins relieve the cell shrinkage upon hyperosmosis.
5) CAHS proteins increase the cell survival against hyperosmotic stress.

Again, we agree that ‘cytoskeleton-like’ is a relatively indirect claim. On the other hand, we think that the ‘stress-dependent cell stiffening’ by CAHS proteins is directly supported by the experimental evidence. Hence, we would like to propose the new title as ‘Stress-dependent cell-stiffening by tardigrade tolerance proteins reversibly forming filamentous network and gel’. All aspects in the new title are directly supported by the experimental evidence also not claiming the causal relationships among the cell-stiffening, the formation of filamentous network and the gel transition.
We are glad to discuss about this point further if necessary.

As you address these items, please take this last chance to review your reference list to ensure that it is complete and correct. If you have cited papers that have been retracted, please include the rationale for doing so in the manuscript text, or remove these references and replace them with relevant current references. Any changes to the reference list should be mentioned in the cover letter that accompanies your revised manuscript.
Accordingly, we have carefully reviewed our reference list in the manuscript and confirmed they are complete and correct without retraction. Thus, there is no change in our reference list.

DATA POLICY:

Thank you for providing the data underlying the graphs shown in the figures. However, we are missing the data from some of them:
Fig. 1C-E, G; Fig. S20, Fig. S24B and Fig. S25A
** Please add them to the data file or let us know where can we find the data. In addition, the labels shown in Fig. 4D data seem to be in Chinese, thus please translate them into English.*
Thank you so much for a careful check and we apologize for the presence of non-English

(Japanese/Chinese) letters. We have added the underlying numerical data in S4_Data for Fig. 1C-D, G; Fig. S20; Fig. S24B and Fig. S25A; and in S2_Data for Fig. 1E. We have also translated the explanation to English in Fig. 4D data in S4_Data.
In addition, we have also added the numerical data in S4_Data for Fig. 3C, S2, S3 and S17. We also improved the readability of S4_Data.

** Please also ensure that figure legends in your manuscript include information ON WHERE THE UNDERLYING DATA CAN BE FOUND.*
We have added the description in the figure legends about where the corresponding data is available, e.g., "The underlying numerical data are available in S4 Data (C, D and G), in S2 Data (E) and in S1 Data (F)." in the legend of Figure 1 and so on.

** Please make sure that the data you have deposited in the jPOST repository of ProteomeXchange Consortium (ID: PXD030241) is made publicly available at this stage.*
We have made the proteome data publicly available, which we have deposited in the jPOST reposity (ID: PXD030241). The data can be retrieved at the URL, http://proteomecentral.proteomexchange.org/cgi/GetDataset?ID=PXD030241 or https://repository.jpostdb.org/entry/JPST001416.
We have also added the description about the URL as "The mass spectrometry proteomics data have been deposited to the ProteomeXchange Consortium via the jPOST repository with the dataset identifier PXD030241 and can be retrieved at http://proteomecentral.proteomexchange.org/cgi/GetDataset?ID=PXD030241." in the corresponding Methods section (p.23, ls.584-585).
