# Supplementary material for: Stress-dependent cell stiffening by tardigrade tolerance proteins that reversibly form a filamentous network and gel
Source: PLoS Biol. 2022 Sep 6;20(9):e3001780. doi: 10.1371/journal.pbio.3001780 (PMC9592077; doi:10.1371/journal.pbio.3001780)
Supplement: Peer review history

# Editor report: Decision Letter 0

**DOI:** 10.1371/journal.pbio.3001780.r001

**Authors:** Ines Alvarez-Garcia

2 Oct 2021

Dear Dr Kunieda, 

Thank you for submitting your manuscript entitled "Stress-dependent dynamic and reversible formation of cytoskeleton-like filaments and gel-transition by tardigrade tolerance proteins" for consideration as a Research Article by PLOS Biology.

Your manuscript has now been evaluated by the PLOS Biology editorial staff as well as by an academic editor with relevant expertise and I am writing to let you know that we would like to send your submission out for external peer review.

However, before we can send your manuscript to reviewers, we need you to complete your submission by providing the metadata that is required for full assessment. To this end, please login to Editorial Manager where you will find the paper in the 'Submissions Needing Revisions' folder on your homepage. Please click 'Revise Submission' from the Action Links and complete all additional questions in the submission questionnaire.

Once your full submission is complete, your paper will undergo a series of checks in preparation for peer review. Once your manuscript has passed the checks it will be sent out for review. 

If your manuscript has been previously reviewed at another journal, PLOS Biology is willing to work with those reviews in order to avoid re-starting the process. Submission of the previous reviews is entirely optional and our ability to use them effectively will depend on the willingness of the previous journal to confirm the content of the reports and share the reviewer identities. Please note that we reserve the right to invite additional reviewers if we consider that additional/independent reviewers are needed, although we aim to avoid this as far as possible. In our experience, working with previous reviews does save time. 

If you would like to send your previous reviewer reports to us, please specify this in the cover letter, mentioning the name of the previous journal and the manuscript ID the study was given, and include a point-by-point response to reviewers that details how you have or plan to address the reviewers' concerns. Please contact me at the email that can be found below my signature if you have questions. 

Please re-submit your manuscript within two working days, i.e. by Oct 05 2021 11:59PM.

Login to Editorial Manager here: https://www.editorialmanager.com/pbiology

During resubmission, you will be invited to opt-in to posting your pre-review manuscript as a bioRxiv preprint. Visit http://journals.plos.org/plosbiology/s/preprints for full details. If you consent to posting your current manuscript as a preprint, please upload a single Preprint PDF when you re-submit. 

Given the disruptions resulting from the ongoing COVID-19 pandemic, please expect delays in the editorial process. We apologise in advance for any inconvenience caused and will do our best to minimize impact as far as possible.

Feel free to email us at plosbiology@plos.org if you have any queries relating to your submission.

Kind regards,

Ines

--

Ines Alvarez-Garcia, PhD

Senior Editor

PLOS Biology

ialvarez-garcia@plos.org

# Peer review: Decision Letter 1

**DOI:** 10.1371/journal.pbio.3001780.r002

**Authors:** Ines Alvarez-Garcia

29 Nov 2021

Dear Takekazu,

Thank you for submitting your manuscript entitled "Stress-dependent dynamic and reversible formation of cytoskeleton-like filaments and gel-transition by tardigrade tolerance proteins" for consideration as a Research Article at PLOS Biology. Thank you also for your patience as we completed our editorial process, and please accept my apologies for the delay in providing you with our decision. Your manuscript has been evaluated by the PLOS Biology editors, an Academic Editor with relevant expertise, and by two independent reviewers.

As you will see, the reviewers are positive and find the conclusions of your manuscript interesting, however they also raise several points that would need to be addressed for us to consider the paper further for publication. Reviewer 1 thinks that you should demonstrate that CAHS3/12 form cytoskeletal fibres and function as temporary cytoskeleton, and suggests an experiment using Drosophila S2 cells to demonstrate this point. Reviewer 2 asks for several clarifications, including the fact that CAHS proteins are only present in eutardigrades and whether or not the concentration used for the CAHS proteins is physiologically relevant. After consulting with the Academic Editor, we have decided to invite a revision and that demonstrating the point raised by Reviewer 1 on the cytoskeletal function of the CASH proteins is key for the publication of the manuscript.

In light of the reviews (attached below), we will not be able to accept the current version of the manuscript, but we would welcome re-submission of a revised version that takes into account the reviewers' comments. We cannot make any decision about publication until we have seen the revised manuscript and your response to the reviewers' comments. Your revised manuscript is also likely to be sent for further evaluation by the reviewers.

We expect to receive your revised manuscript within 3 months. 

Please email us (plosbiology@plos.org) if you have any questions or concerns, or would like to request an extension. At this stage, your manuscript remains formally under active consideration at our journal; please notify us by email if you do not intend to submit a revision so that we may end consideration of the manuscript at PLOS Biology.

**IMPORTANT - SUBMITTING YOUR REVISION**

Your revisions should address the specific points made by each reviewer. Please submit the following files along with your revised manuscript:

1. A 'Response to Reviewers' file - this should detail your responses to the editorial requests, present a point-by-point response to all of the reviewers' comments, and indicate the changes made to the manuscript. 

*NOTE: In your point by point response to the reviewers, please provide the full context of each review. Do not selectively quote paragraphs or sentences to reply to. The entire set of reviewer comments should be present in full and each specific point should be responded to individually, point by point.

You should also cite any additional relevant literature that has been published since the original submission and mention any additional citations in your response. 

2. In addition to a clean copy of the manuscript, please also upload a 'track-changes' version of your manuscript that specifies the edits made. This should be uploaded as a "Related" file type. 

3. Resubmission Checklist

When you are ready to resubmit your revised manuscript, please refer to this resubmission checklist: https://plos.io/Biology_Checklist

To submit a revised version of your manuscript, please go to https://www.editorialmanager.com/pbiology/ and log in as an Author. Click the link labelled 'Submissions Needing Revision' where you will find your submission record. 

Please make sure to read the following important policies and guidelines while preparing your revision and fulfil the editorial requests:

a) *PLOS Data Policy*

Please note that as a condition of publication PLOS' data policy (http://journals.plos.org/plosbiology/s/data-availability) requires that you make available all data used to draw the conclusions arrived at in your manuscript. If you have not already done so, you must include any data used in your manuscript either in appropriate repositories, within the body of the manuscript, or as supporting information (N.B. this includes any numerical values that were used to generate graphs, histograms etc.). Please also indicate in each figure legend where the data can be found. For an example see here: http://www.plosbiology.org/article/info%3Adoi%2F10.1371%2Fjournal.pbio.1001908#s5

b) *Published Peer Review*

Please note while forming your response, if your article is accepted, you may have the opportunity to make the peer review history publicly available. The record will include editor decision letters (with reviews) and your responses to reviewer comments. If eligible, we will contact you to opt in or out. Please see here for more details:

https://blogs.plos.org/plos/2019/05/plos-journals-now-open-for-published-peer-review/

c) *Blot and Gel Data Policy*

Please provide the original, uncropped and minimally adjusted images supporting all blot and gel results reported in an article's figures or Supporting Information files. We will require these files before a manuscript can be accepted so please prepare them now, if you have not already uploaded them. Please carefully read our guidelines for how to prepare and upload this data: https://journals.plos.org/plosbiology/s/figures#loc-blot-and-gel-reporting-requirements

d) *Blurb*

Please also provide a blurb which (if accepted) will be included in our weekly and monthly Electronic Table of Contents, sent out to readers of PLOS Biology, and may be used to promote your article in social media. The blurb should be about 30-40 words long and is subject to editorial changes. It should, without exaggeration, entice people to read your manuscript. It should not be redundant with the title and should not contain acronyms or abbreviations. For examples, view our author guidelines: https://journals.plos.org/plosbiology/s/revising-your-manuscript#loc-blurb

e) *Protocols deposition*

To enhance the reproducibility of your results, we recommend that if applicable you deposit your laboratory protocols in protocols.io, where a protocol can be assigned its own identifier (DOI) such that it can be cited independently in the future. For instructions see: https://journals.plos.org/plosbiology/s/submission-guidelines#loc-materials-and-methods

Thank you again for your submission to our journal. We hope that our editorial process has been constructive thus far, and we welcome your feedback at any time. Please don't hesitate to contact us if you have any questions or comments.

Sincerely,

Ines

--

Ines Alvarez-Garcia, PhD

Senior Editor

PLOS Biology

ialvarez-garcia@plos.org

------------------------------------------------------------------

Reviewers' comments

Rev. 1:

Tanaka et al. reports reversible stress-induced fiber formation of CAHS proteins, which are tardigrade-unique abundant heat soluble proteins presumed to relate to their anhydrobiotic capabilities. They propose that these set of proteins are novel stress-dependent cytoskeletal proteins. The paper is nicely written, and experimental designs are very comprehensive and thorough, and the beautiful microscope images of filaments in human culture cells are convincing.

I have several comments to possibly improve the paper as detailed below.

1. The authors firstly screen for reversibly soluble proteins using desiccation-mimicking condition with TFE, and name this "Dehydration-dependent reversibly condensing proteins (DRPs)".

Firstly, I would like to suggest a different naming to avoid confusion with DRPs (dynamin-related proteins including Drp1, Dnm1, Dlp1 and so on) that are also frequently involved in stress response. Secondly, I am not certain wether the use of TFE actually mimicks "dehydration". TFE is known as an alpha-inducer, and this may be affecting the type of proteins, and not necessarity dehydration-dependence. As explained thoroughly in the manuscript, CAHS proteins are enriched in helical structures, and other enriched proteins such as actin as well as peroxiredoxins contain large percentage of alpha-helices. I understand that the condition may be too harsh on most proteins, but simply desiccating extracted proteins and rehydrating them could be a more direct protocol to obtain actual "dehydration-dependent reversibly condensing" proteins, and am curious if the current method with TFE actually recapitulates same set of proteins. This is important, since the authors discuss rather extensively that their method with TFE would see wider potential applications in enriching "dehydration-dependent" proteins.

2. In the introduction, after stressing the importance of Intermediate Filament (IF), the authors state "Tardigrades have actin filaments and microtubles, but largely lack cytoplasmic IFs, except a tardigrade-unique IF protein called cytotardin". This is a bit misleading, because the lack of canonical cytoplasmic IFs is a panarthropoda feature. There are many anhydrobiotic arthropods, so please make it clear that this is not tardigrade-unique loss.

3. Recently, two papers on a very similar topic have been published, and another preprint is available, publication of two of which predates the submission of this paper.

Yagi-Utsumi et al. (2021) Sci Rep. reports CAHS1 (R. varieornatus) to reversibly form filaments and gels in vitro, and condensates in human cells, and that the C-terminal region is essential for such fiber formation.

Malki et al. (2021) Angewandte Chemie reports CAHS8 (H. exemplaris) to reversibly form filaments and gels in vitro.

I think the current manuscript still has its own significance, but nevertheless discussions regarding similarities and differences of these closely related works would strengthen the presented arguments.

4. The authors use HEp-2 cells in this work. They also use Drosophila S2 cells, so the observed fibrilation is not cell-line specific, but I am curious for the choice of this cell line, because the previous works by the authors' lab consistently utilized HEK293T cells (for example, Yamaguchi et al. 2012, Tanaka et al. 2015, and Hashimoto et al. 2016). It would be interesting to compare the differences if somehow fiber formation was not feasible in HEK293T.

5. The authors show fibril formation in Drosophila S2 cells, which is convincing. I think this is a perfect opportunity to test the actual cytoskeletal function of CAHS (if there actually is), since the current paper provides no evidence regarding actual protective role of CAHS upon dehydration or stress-induction. Since Drosophila also lack IFs like tardigrades, was there any phenotypic differences regarding tolerance in S2 cells expressing CAHS? It would really strenghen the argument if the authors can actually show that these proteins "effectively counteract the deformative forces" (Line 104).

Minor comments:

Proteomic analysis data could be deposited to one of ProteomExchange repositories.

Rev. 2:

This manuscript deals with the putative function of the newly discovered CAHS proteins, which belong to the so-called "tardigrade unique proteins" found exclusively among eutardigrades. The manuscript is well-written and based on thoroughly conducted experiments. Please find my more detailed comments below.

Conclusions are generally supported by the data. My only major concern is that you over interpret your data, when you neglect to mention that CAHS proteins are missing in one of the two major evolutionary lineages within the tardigrades. In other words, as CAHS proteins are found exclusively among eutardigrades, they may not be as important for anhydrobiosis as perceived from reading your manuscript. Consequently, I recommend that you briefly discuss papers showing that anhydrobiotic heterotardigrades lack CAHS proteins (Kamilari et al. 2019. BMC Genomics 20, 607; Murai et al. 2021. BMC Genomics 22, 813) and that you avoid statements such as "….(CAHS) proteins which are essential for the anhydrobiotic survival of tardigrades". The latter and other comparable statements are simply not in accord with current knowledge within the field. Along this line, I suggest that you in the title and throughout the abstract, introduction etc. replace the word "tardigrade" with the more accurate term "eutardigrade".

You show that cytoskeletal proteins, such as actin, are significantly enriched in DRPs. I lack a comment on the possible significance of well-known cytoskeletal proteins in relation to stabilization of tardigrade cell structure during desiccation.

In the introduction (and discussion) you briefly discuss and dismiss the possibility that CAHS proteins contribute to vitriﬁcation of the cytoplasm during desiccation. It would be interesting if you could present a more elaborate comparison between vitrification and the gel-transition observed in the current manuscript. What happens to the CAHS hydrogels, if exposed to very low relative humidity (and extremely low temperatures or pressures)?

You state that "In vitro gel transition was observed when using a relatively high concentration (~ 4 mg/mL) of CAHS solution". The manuscript would benefit from a discussion on whether the used concentrations of CAHS proteins are physiological relevant. What are the presumed concentrations of the proteins within cells of Ramazzottius varieornatus in the active and desiccated states?

Please carefully check figures and text for minor errors. E.g. correct to "Relative" on y-axis in Fig 2C and explain white arrowheads in Fig 2F. The images of S2 cells presented in S8 Fig seem to be of low quality.

# Author comment: Author response to Decision Letter 1

**DOI:** 10.1371/journal.pbio.3001780.r003

6 Mar 2022

Attachment Submitted filename: Response_to_reviewers_20220304.pdf Click here for additional data file.

# Peer review: Decision Letter 2

**DOI:** 10.1371/journal.pbio.3001780.r004

**Authors:** Ines Alvarez-Garcia

29 Apr 2022

Dear Dr Kunieda,

Thank you for submitting a revised version of your manuscript entitled "Stress-dependent cell stiffening by tardigrade tolerance proteins through reversible formation of cytoskeleton-like filamentous network and gel-transition​" for consideration as a Research Article at PLOS Biology. Please accept my apologies for the delay in providing you with our decision. This revised version of your manuscript has been evaluated by the PLOS Biology editors, the Academic Editor and one of the original reviewers.

You will see that the reviewer is positive and appreciates the significant improvements done in the manuscript. However, the reviewer still raises two issues that remain to be addressed, one regarding the misunderstanding of the alpha-inducing property of TFE and the second one on the fact that s/he is not fully convinced that the CAHS3/12 fibres are ‘cystoskeletal’, given that the control experiment is an empty vector and not non-fibre producing CAHS. After discussing these issues with the Academic Editor, we think you should address the remaining issues raised. In addition, we believe that given the nature of the findings the manuscript would be a better fit as a Discovery Report, thus we would like you to adapt the manuscript to that format (https://journals.plos.org/plosbiology/s/what-we-publish#loc-discovery-report).

In light of the reviews (attached below), we are pleased to offer you the opportunity to address the remaining points from the reviewer in a revised version that we anticipate should not take you very long. We will then assess your revised manuscript and your response to the reviewers' comments and we may consult the reviewers again.

We expect to receive your revised manuscript within 1 month.

Please email us (plosbiology@plos.org) if you have any questions or concerns, or would like to request an extension. At this stage, your manuscript remains formally under active consideration at our journal; please notify us by email if you do not intend to submit a revision so that we may end consideration of the manuscript at PLOS Biology.

**IMPORTANT - SUBMITTING YOUR REVISION**

Your revisions should address the specific points made by each reviewer. Please submit the following files along with your revised manuscript:

1. A 'Response to Reviewers' file - this should detail your responses to the editorial requests, present a point-by-point response to all of the reviewers' comments, and indicate the changes made to the manuscript. 

*NOTE: In your point by point response to the reviewers, please provide the full context of each review. Do not selectively quote paragraphs or sentences to reply to. The entire set of reviewer comments should be present in full and each specific point should be responded to individually.

You should also cite any additional relevant literature that has been published since the original submission and mention any additional citations in your response. 

2. In addition to a clean copy of the manuscript, please also upload a 'track-changes' version of your manuscript that specifies the edits made. This should be uploaded as a "Related" file type. 

*Resubmission Checklist*

When you are ready to resubmit your revised manuscript, please refer to this resubmission checklist: https://plos.io/Biology_Checklist

To submit a revised version of your manuscript, please go to https://www.editorialmanager.com/pbiology/ and log in as an Author. Click the link labelled 'Submissions Needing Revision' where you will find your submission record. 

Thank you again for your submission to our journal. We hope that our editorial process has been constructive thus far, and we welcome your feedback at any time. Please don't hesitate to contact us if you have any questions or comments.

Sincerely,

Ines

--

Ines Alvarez-Garcia, PhD

Senior Editor

PLOS Biology

ialvarez-garcia@plos.org

------------------------------------------------------------------

Reviewers' comments

Rev. 1:

The authors have put an extensive effort in revising the manuscript, and the addition of new experiment using S2 cells is fantastic in showing that the expression of CAHS3 in heterologous condition confers osmotic stress tolerance. Induction or enhancement of desiccation tolerance in heterologous cells has been reported by Boothby et al. (2017) Mol Cell, but the survival rate was extremely low in the above work, so a more definitive confirmation in a host more closely related to tardigrades is an important progress.

I appreciate the consideration in changing the name of DRYPs to avoid confusion. The authors, however, seems to misunderstand the fact regarding the alpha-inducing property of TFE. The authors state in the cover letter "TFE is known to induce the conformational changes (largely helix as commented by the reviewer) of several desiccation-tolerance proteins (e.g., LEA proteins or CAHS proteins) similarly as in a dehydrated condition." and "Dehydration could induce conformational change to helix-rich structure of some unstructured proteins and TFE might mimic this conformational change." But in fact TFE is a well-known alpha-inducer commonly used in protein engineering, not specific to "desiccation-tolerance proteins", which stablizes the alhpa-helical structure but not the beta-region (there are numerous papers, but see for example, Shiraki et al. 1995 JMB). My original concern (which remains to be addressed) is that "Desolvation-induced ReversiblY condensing Proteins (DRYPs)" proposed may actually be "Alpha-induction-based Reversibly condensing proteins" and may not necessarily mimic desiccation. I understand, and the data in Fig.1 nicely shows, that there is an overlap - CAHS and LEA happen to be good alpha-formers and get enriched - but this may be a partial picture of desiccation treatment, and there is still little evidence that desiccation itself actually promotes alpha-helical structures universally in the proteins. Therefore, while I understand the potential of this method, I am not fully convinced for the validity to call it "Desolvation-...", and even if so, limitation of this method should be discussed, that it may significantly overlook structural changes other than those in alpha-helix, and that there could be artefacts related to the alpha-inducing nature of TFE (which might not necessarily happen upon desiccation universally in all proteins).

Again I congratulate the authors on the additional work in Fig.9 regarding the osmotic stress tolerance of S2 cells. It nicely adds a convincing evidence that these heat-soluble proteins actually contribute to desiccation tolerance in cells. I, however, still am not fully convinced that the CAHS3/12 fibers are "cytoskeletal", since the control experiment is only an empty vector and not non-fiber producing CAHS (like CAHS8 or truncated CAHS3 or 12). Increase in osmotic stress tolerance in non-fiber producing LEA-family proteins are reported elsewhere (Hibshman and Goldstein 2021 BMC Biology, or the authors' own work Tanaka et al. 2015 PLoS One), so in order to confirm that the filamentation is essential and "cytoskeletal", it requires comparison to non-"cytoskeletal" gels and significant higher tolerance over them.

Yet again, I think the current work nicely demonstrates with beautiful microscopy that these proteins actually form filaments in cells, and I find the discussion and speculation that these proteins serve putative functions like cytoskeletons inspiring. However, I still do not think there is strong evidence for the cytoskeletal functionality, to include and signify it in the title.

Minor comment:

pg.20 Line 506 "although the necessity of such helix structure for filament/gel formation was not demonstrated. " While I agree that the analysis is not as detailed, I think Yagi-Utsumi et al. (2021) Figure S1 rather clearly show the contribution of the conserved helical C-terminal half is essential in filament/gel formation both by NMR and AFM analyses.

# Author comment: Author response to Decision Letter 2

**DOI:** 10.1371/journal.pbio.3001780.r005

3 Jun 2022

Attachment Submitted filename: Response_to_reviewers_20220602.pdf Click here for additional data file.

# Editor report: Decision Letter 3

**DOI:** 10.1371/journal.pbio.3001780.r006

**Authors:** Ines Alvarez-Garcia

8 Jul 2022

Dear Dr Kunieda,

Thank you for your patience while we considered your revised manuscript entitled "Stress-dependent cell stiffening by tardigrade tolerance proteins reversibly forming cytoskeleton-like filamentous network and gel" for publication as a Discovery Report at PLOS Biology. This revised version of your manuscript has been evaluated by the PLOS Biology editors, and the Academic Editor.

Based on our Academic Editor's assessment of your revision, we are likely to accept this manuscript for publication, provided you satisfactorily address the following data and other policy-related requests (see below).

In addition, we would like you to have a careful look again through the manuscript and make sure that the data are correctly presented (specially the statistics) and referred to in the text. Given the extensive reorganization of the figures and the large number of supplemental figures, it is easy for mistakes/omissions to occur. We would also like you to consider a suggestion to improve the title:

“Tardigrade cytoskeleton-like proteins can reversibly form a filamentous network and undergo gel-transition in response to stress"

As you address these items, please take this last chance to review your reference list to ensure that it is complete and correct. If you have cited papers that have been retracted, please include the rationale for doing so in the manuscript text, or remove these references and replace them with relevant current references. Any changes to the reference list should be mentioned in the cover letter that accompanies your revised manuscript.

We expect to receive your revised manuscript within two weeks. 

To submit your revision, please go to https://www.editorialmanager.com/pbiology/ and log in as an Author. Click the link labelled 'Submissions Needing Revision' to find your submission record. Your revised submission must include the following:

- a cover letter that should detail your responses to any editorial requests, if applicable, and whether changes have been made to the reference list

- a Response to Reviewers file that provides a detailed response to the reviewers' comments (if applicable)

- a track-changes file indicating any changes that you have made to the manuscript. 

NOTE: If Supporting Information files are included with your article, note that these are not copyedited and will be published as they are submitted. Please ensure that these files are legible and of high quality (at least 300 dpi) in an easily accessible file format. For this reason, please be aware that any references listed in an SI file will not be indexed. For more information, see our Supporting Information guidelines:

https://journals.plos.org/plosbiology/s/supporting-information

*Published Peer Review History*

Please note that you may have the opportunity to make the peer review history publicly available. The record will include editor decision letters (with reviews) and your responses to reviewer comments. If eligible, we will contact you to opt in or out. Please see here for more details:

https://blogs.plos.org/plos/2019/05/plos-journals-now-open-for-published-peer-review/

*Press*

Should you, your institution's press office or the journal office choose to press release your paper, please ensure you have opted out of Early Article Posting on the submission form. We ask that you notify us as soon as possible if you or your institution is planning to press release the article.

*Protocols deposition*

To enhance the reproducibility of your results, we recommend that if applicable you deposit your laboratory protocols in protocols.io, where a protocol can be assigned its own identifier (DOI) such that it can be cited independently in the future. Additionally, PLOS ONE offers an option for publishing peer-reviewed Lab Protocol articles, which describe protocols hosted on protocols.io. Read more information on sharing protocols at https://plos.org/protocols?utm_medium=editorial-email&utm_source=authorletters&utm_campaign=protocols

Please do not hesitate to contact me should you have any questions.

Sincerely,

Ines

--

Ines Alvarez-Garcia, PhD

Senior Editor,

PLOS Biology

ialvarez-garcia@plos.org

------------------------------------------------------------------------

DATA POLICY:

Thank you for providing the data underlying the graphs shown in the figures. However, we are missing the data from some of them:

Fig. 1C-E, G; Fig. S20, Fig. S24B and Fig. S25A

* Please add them to the data file or let us know where can we find the data. In addition, the labels shown in Fig. 4D data seem to be in Chinese, thus please translate them into English.

* Please also ensure that figure legends in your manuscript include information ON WHERE THE UNDERLYING DATA CAN BE FOUND.

* Please make sure that the data you have deposited in the jPOST repository of ProteomeXchange Consortium (ID: PXD030241) is made publicly available at this stage.

# Author comment: Author response to Decision Letter 3

**DOI:** 10.1371/journal.pbio.3001780.r007

14 Jul 2022

Attachment Submitted filename: response_to_requests20220713.pdf Click here for additional data file.

# Editor report: Decision Letter 4

**DOI:** 10.1371/journal.pbio.3001780.r008

**Authors:** Ines Alvarez-Garcia

2 Aug 2022

Dear Dr Kunieda,

Thank you for the submission of your revised Discovery Report entitled "Stress-dependent cell-stiffening by tardigrade tolerance proteins reversibly form a filamentous network and gel" for publication in PLOS Biology. On behalf of my colleagues and the Academic Editor, Carole Parent, I am happy to say that we can in principle accept your manuscript for publication, provided you address any remaining formatting and reporting issues. These will be detailed in an email you should receive within 2-3 business days from our colleagues in the journal operations team; no action is required from you until then. Please note that we will not be able to formally accept your manuscript and schedule it for publication until you have completed any requested changes.

Please take a minute to log into Editorial Manager at http://www.editorialmanager.com/pbiology/, click the "Update My Information" link at the top of the page, and update your user information to ensure an efficient production process.

PRESS

We frequently collaborate with press offices. If your institution or institutions have a press office, please notify them about your upcoming paper at this point, to enable them to help maximise its impact. If the press office is planning to promote your findings, we would be grateful if they could coordinate with biologypress@plos.org. If you have previously opted in to the early version process, we ask that you notify us immediately of any press plans so that we may opt out on your behalf.

We also ask that you take this opportunity to read our Embargo Policy regarding the discussion, promotion and media coverage of work that is yet to be published by PLOS. As your manuscript is not yet published, it is bound by the conditions of our Embargo Policy. Please be aware that this policy is in place both to ensure that any press coverage of your article is fully substantiated and to provide a direct link between such coverage and the published work. For full details of our Embargo Policy, please visit http://www.plos.org/about/media-inquiries/embargo-policy/.

Thank you again for choosing PLOS Biology for publication and supporting Open Access publishing. We look forward to publishing your study. 

Sincerely, 

Ines

--

Ines Alvarez-Garcia, PhD

Senior Editor

PLOS Biology

ialvarez-garcia@plos.org
